# Rapid construction of metabolite biosensors using domain-insertion profiling

**DOI:** 10.1038/ncomms12266

**Published:** 2016-07-29

**Authors:** Dana C. Nadler, Stacy-Anne Morgan, Avi Flamholz, Kaitlyn E. Kortright, David F. Savage

**Affiliations:** 1 Department of Molecular & Cell Biology, University of California, Berkeley, California 94720, USA; 2 Department of Chemistry, University of California, Berkeley, California 94720, USA; 3 Energy Biosciences Institute, University of California, Berkeley, California 94720, USA

## Abstract

Single-fluorescent protein biosensors (SFPBs) are an important class of probes that enable the single-cell quantification of analytes *in vivo*. Despite advantages over other detection technologies, their use has been limited by the inherent challenges of their construction. Specifically, the rational design of green fluorescent protein (GFP) insertion into a ligand-binding domain, generating the requisite allosteric coupling, remains a rate-limiting step. Here, we describe an unbiased approach, termed domain-insertion profiling with DNA sequencing (DIP-seq), that combines the rapid creation of diverse libraries of potential SFPBs and high-throughput activity assays to identify functional biosensors. As a proof of concept, we construct an SFPB for the important regulatory sugar trehalose. DIP-seq analysis of a trehalose-binding-protein reveals allosteric hotspots for GFP insertion and results in high-dynamic range biosensors that function robustly *in vivo*. Taken together, DIP-seq simultaneously accelerates metabolite biosensor construction and provides a novel tool for interrogating protein allostery.

## Abstract (web summary)

In the construction of single fluorescent protein biosensors, selection of the insertion point of a fluorescent protein into a ligand-binding domain is a rate-limiting step. Here, the authors develop an unbiased, high-throughput approach, called domain insertion profiling with DNA sequencing (DIP-seq), to generate a novel trehalose biosensor.

## Graphical abstract

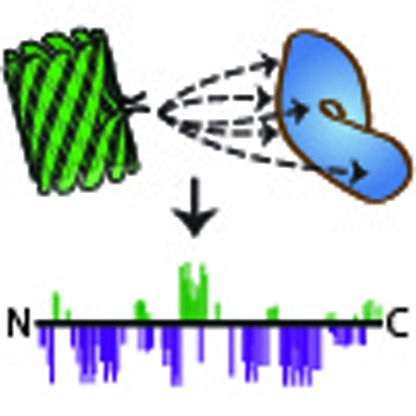

Probing metabolism *in situ* is critical to understanding the molecular physiology of the cell^1^,^2^. Current analytical techniques, such as mass spectrometry, have proven extremely informative but are also limited by their low spatial resolution, low throughput, and invasiveness^3^. Alternatively, an expanding array of genetically encoded biosensors allows quantification of individual metabolites with high spatiotemporal resolution in the native cellular context^3^,^4^,^5^. These protein- and RNA-based biosensors detect metabolites with exquisite specificity and trigger real-time fluorescent or chemiluminescent output signals.

Single-fluorescent protein biosensors (SFPBs) are particularly promising for their combination of linear response to substrate concentration and high dynamic range. SFPBs are composed of a circularly permuted green fluorescent protein (cpGFP) inserted into the primary sequence of a specific ligand-binding domain (LBD) (Fig. 1a)^6^. Allosteric coupling between the LBD and cpGFP domains engenders a ligand-dependent fluorescence change on metabolite binding. For example, the GCaMP family of genetically encoded calcium indicators is constructed by inserting cpGFP between calmodulin and the M13 peptide^7^. However, despite their potential, the palette of existing SFPBs is limited due to the difficulty of rationally designing an allosteric connection between cpGFP and a given LBD.

Randomized, library-based approaches have successfully created functional allosteric linkages between protein domains, but have not been applied to biosensors^8^. Compared with the rational design of a few carefully selected fusions, random domain-insertion methods are advantageous because they potentially sample all possible insertion-site variants without prior structural or mechanistic knowledge. This mimics natural gene fusion, the mechanism used by evolution to generate modular, multi-domain proteins^9^.

Here, motivated by recent work exploring the sequence-function space of proteins in a high-throughput fashion^10^,^11^, we describe a library-based approach, incorporating fluorescent screening and next-generation sequencing (NGS), that aims to identify allosteric hotspots as a means of accelerating the development of protein biosensors. It was previously shown that *in vitro* transposition can be used to randomly insert one protein domain into another^12^,^13^. We have refined this approach to improve efficiency and minimize the transposon ‘scar' sequence in fusion proteins. Using the *Escherichia coli* D-maltose-binding protein (MBP)^14^ as a proof of concept, we demonstrate that transposon-mediated insertion of cpGFP creates diverse domain-insertion libraries containing allosterically coupled biosensors. To further enhance our method for generating SFPBs, we construct a library of cpGFP insertions within the *Thermococcus litoralis* D-trehalose/D-maltose-binding protein (TMBP)^15^, subject it to successive rounds of functional enrichment using fluorescence-activated cell sorting (FACS), and monitor this process using DNA sequencing (Fig. 1b). Tracking of the libraries with NGS reveals that enrichment correlates closely with biosensor function, which leads to the facile isolation of high-dynamic-range biosensors that function well *in vivo*. Taken together, domain-insertion profiling with sequencing (DIP-seq) allows for the rapid construction of functional SFPBs and, more broadly, the identification of allosteric hotspots within any protein.

## Results

### Creating random domain-insertion libraries using transposons

**Figure 1:**
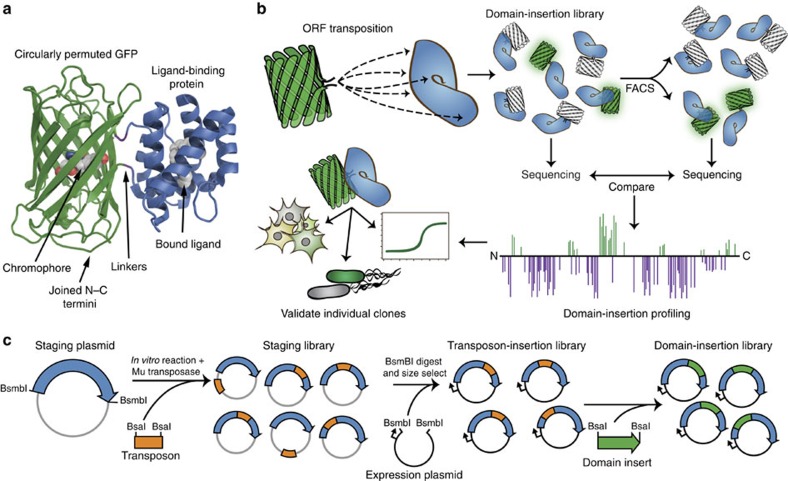
High-throughput biosensor construction using DIP-seq. (**a**) Illustration of a single-FP metabolite protein biosensor. cpGFP is fused to a LBD in a manner such that metabolite binding by the LBD causes a change in fluorescence of the attached cpGFP. (**b**) Overview of the domain-insertion profiling method used to create and identify functional biosensors. A diverse library of fusions, with cpGFP inserted into a LBD, is created and screened with FACS. Initial and sorted libraries undergo NGS analysis and these data are used to identify insertion sites within the LBD that are enriched during screening. Clones of interest are individually tested to validate biosensor functionality. (**c**) Method of domain-insertion library creation. An engineered transposon containing a selectable marker is inserted into a staging plasmid carrying the LBD ORF using an *in vitro* transposase reaction. Staging plasmids with an insertion are selected for, purified and digested with an enzyme that releases the LBD ORF from the staging backbone (grey). LBD ORFs with an inserted transposon are size-selected and cloned into an expression plasmid (black). Finally, a domain of interest (in this paper, cpGFP) is inserted into the cloning site created by the modified transposon.

A method for randomized insertion of one protein domain into another was developed using transposons. Synthetic Mu transposition into plasmid DNA can be catalysed *in vitro* (refs 16, 17) and so a number of Mu variants, possessing internal DNA type IIS restriction sites, was assayed for function (Supplementary Fig. 1). The location of these sites provided programmable cut sites with minimal scarring. One variant with BsaI sites (Mu-BsaI), encoding alanine–serine linkers at either end, was used to introduce insertions throughout our target LBDs (Fig. 1c and Supplementary Fig. 1c). After transposition, the open reading frame (ORF) insertion library was subcloned into a new expression vector to eliminate insertions outside of the target ORF. The Mu-BsaI cassette was then excised and replaced with cpGFP by Golden Gate assembly with BsaI (Fig. 1c)^18^. This technique provides efficient construction of complete domain-insertion libraries and precise control of connecting linker sequences.

**Figure 2:**
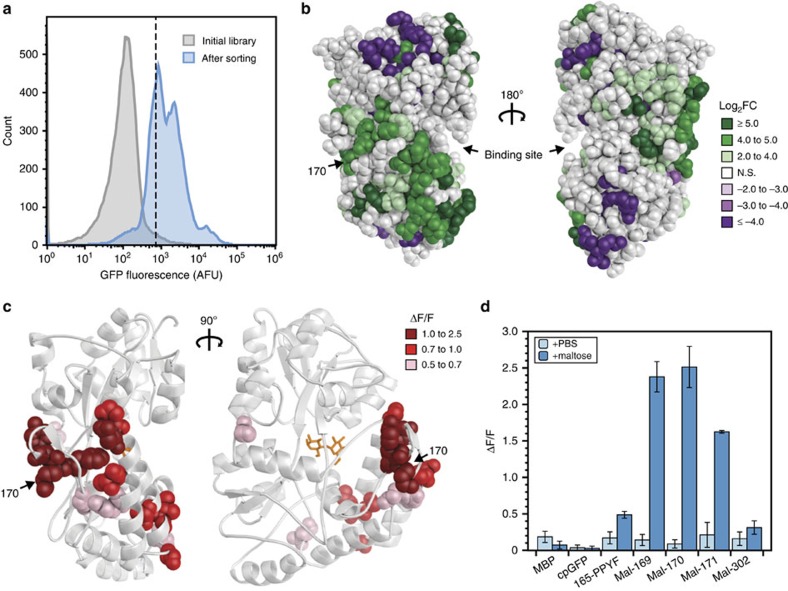
Maltose-binding protein as proof-of-concept target of random domain insertion. (**a**) Fluorescence histograms of representative MBP-cpGFP libraries. Induced libraries were sorted via FACS with gating set to collect cells above a threshold GFP fluorescence level shown with a dashed line. (**b**) Enrichment of cpGFP-insertion sites (log_2_ of fold change) from FACS mapped onto MBP crystal structure (PDB 1ANF). Enrichment calculated from NGS, comparing pre- and post-sort libraries. Insertion sites that went from undetectable to detectable were set at +6, while sites that were cleared from the library (not sequenced in post-sort library) were set at −6. Amino acid 170 is indicated with an arrow. (**c**) Functional biosensor insertion sites mapped onto the MBP structure. Sites highlighted with sphere representation of side chains, with colours showing level of activity (Δ*F*/*F*=(*F*_ligand_−*F*_0_)/*F*_0_). (**d**) Activity assay of individual constructs. Fluorescence change on addition of saturating maltose (1 mM) or PBS was measured, shown by mean±s.d. of Δ*F*/*F* (three biological replicates). Sample 165-PPYF is the previously published construct EcMBP165-cpGFP.PPYF.T203V (ref. 19).

An SFPB fashioned around MBP was used as a proof of concept to validate the utility of domain-insertion libraries. This LBD served as a benchmark for our approach because previous studies used MBP to generate allosteric fusions^8^,^19^. MBP-cpGFP libraries were created in duplicate using the engineered-transposon method. Notably, the initial naive library included insertions in all six reading frames due to the inherent randomness of Mu transposition. Because only productive (forward and in-frame) and correctly folded cpGFP insertions will result in fluorescent constructs, the libraries were screened with FACS in the presence of maltose to deplete unproductive (reverse or out-of-frame) insertions and misfolded constructs (Fig. 2a). NGS sequencing of the sorting process revealed that FACS enriched for forward oriented, in-frame insertions, with 95 productive and only 4 unproductive insertions showing significant enrichment (*P*<0.1). In all, 210 (of 370 possible) productive insertions were detected in the final library (Fig. 2b, Supplementary Fig. 2a,b). These data show that our transposon technique rapidly builds diverse domain-insertion libraries.

Assay of the MBP-cpGFP domain-insertion libraries revealed the presence of several functional maltose biosensors. Constructs from the sorted libraries were expressed and tested for *in vivo* biosensor activity in 96-well plate format. Activity was evaluated by the fluorescent change of cells after addition of saturating maltose to the medium and calculated as Δ*F*/*F* (that is, (*F*_ligand_−*F*_0_)/*F*_0_). Two 96-well plates were screened from each library, and twelve unique cpGFP insertion sites were found to have marked activity (Δ*F*/*F*⩾0.5, Supplementary Fig. 2c). Interestingly, mapping these insertion sites onto the structure of MBP revealed that the sites were three-dimensionally clustered despite being noncontiguous in primary sequence (Fig. 2c). In addition, even though maltose binding causes MBP to close its two domains together in a rigid-body hinge motion^20^,^21^, active insertion sites were not exclusively found in these areas of macroscopic conformational change (Supplementary Fig. 3). These results show that testing a diverse library can locate, and reveal trends of, allosteric hotspots of biosensor function.

Several of these biosensors possessed a high dynamic range (Δ*F*/*F*⩾1). Activity screening revealed that cpGFP insertion at amino acids 169 and 171 produced highly functional biosensors (Δ*F*/*F*=2.4±0.2 and Δ*F*/*F*=1.6±0.1, (mean±s.d.), respectively; Fig. 2d). The intervening site, 170, was not detected in the few plates screened but was found to be enriched in the NGS analysis (Supplementary Fig. 2a). When constructed and assayed, this fusion was shown to be an equally active biosensor (Δ*F*/*F*=2.5±0.3, s.d.; Fig. 2d). These constructs showed greater dynamic range than the best-reported MBP-cpGFP biosensor, EcMBP165-cpGFP.PPYF.T203V (Δ*F*/*F*=0.5±0.1, s.d.; Fig. 2d)^19^. As with previous work, we found that biosensor function could be markedly improved through LBD-cpGFP linker engineering (Δ*F*/*F*=8.1±0.3, s.d.; Supplementary Figs 4 and 5)^22^. Taken together, these experiments with MBP validate the hypothesis that diverse domain-insertion libraries can be used to map allosteric hotspots and construct functional SFPBs.

### DIP-seq robustly generates trehalose biosensors

**Figure 3:**
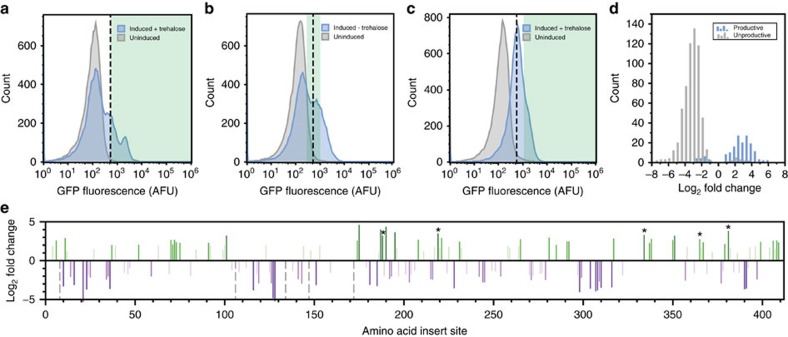
Domain-insertion profiling enriches for fluorescent in-frame proteins. (**a**) Fluorescence histograms of a representative TMBP-cpGFP library during initial sort. Histograms compare uninduced with induced libraries. The induced library was treated and sorted with 1 mM trehalose. (**b**) Fluorescence histograms of sorted library shown in **a** during sort two (that is, sort for low fluorescence in the absence of trehalose). (**c**) Fluorescence histograms of the sorted library from **b** during sort three (that is, high fluorescence in 1 mM trehalose). For **a**–**c**, 25,000 events are shown. The dashed line indicates events at a non-fluorescent sample threshold and green shaded regions indicate gates for sorted cells. (**d**) Histogram of enrichment values, comparing productive (in-frame) insertion constructs and non-productive (out-of-frame and reverse) insertion constructs. Enrichment, shown as log_2_ of the fold change, was calculated with DESeq^47^ from two biological replicates comparing the final library NGS read counts to the initial library. Calculated enrichments with *P*<0.1 shown. (**e**) Profile of enrichment values (initial versus final) for in-frame insertions along TMBP primary sequence. Calculated enrichments with *P*<0.1 shown. Insert sites cleared from the library are represented with gray dashed lines set to −5. Colours are binned for enrichment values, matching Fig. 4c. Asterisks mark isolated and sequenced constructs with activity Δ*F*/*F*⩾0.5 (Supplementary Fig. 9a).

We next tested whether two enhancements could improve our ability to identify the best biosensors and provide a profile of functional domain-insertion sites: (i) additional rounds of FACS, enriching for the desired switching behaviour in the presence and absence of ligand; and (ii) NGS analysis to detect functional biosensors that are enriched but which are in low abundance due to non-uniform libraries. This enhanced approach, which we term DIP-seq, was used to assess the potential of TMBP as a biosensor scaffold for trehalose. Trehalose is a molecule of biological interest that is implicated in stress tolerance and metabolic regulation of yeast and plants but for which there is no available SFPB^23^,^24^,^25^. Independent transposon libraries, in triplicate, were used to create libraries of cpGFP insertions into the TMBP ligand-binding domain. The libraries were then taken through three iterative rounds of FACS screening. An initial round of positive sorting in the presence of trehalose was followed by a negative round without trehalose to enrich for active, switchable biosensors. A final round of positive sorting in the presence of trehalose was conducted to enrich switches still capable of binding trehalose (Fig. 3a–c).

NGS of the library population, in time, revealed FACS applied a strong selective pressure towards constructs with forward, in-frame cpGFP insertions. Two of the libraries were sequenced before and after every sort, and comparison of the final sort to the initial, naive library revealed that productive insertions were broadly enriched while non-productive insertions were strongly depleted (Fig. 3d). Since a large majority of productive insertions were enriched, fold-change values were recalculated relative to only productive insertions for further analysis. These enrichments were mapped to the primary sequence of TMBP and show that TMBP is enriched for insertions throughout its primary sequence (Fig. 3e).

**Figure 4:**
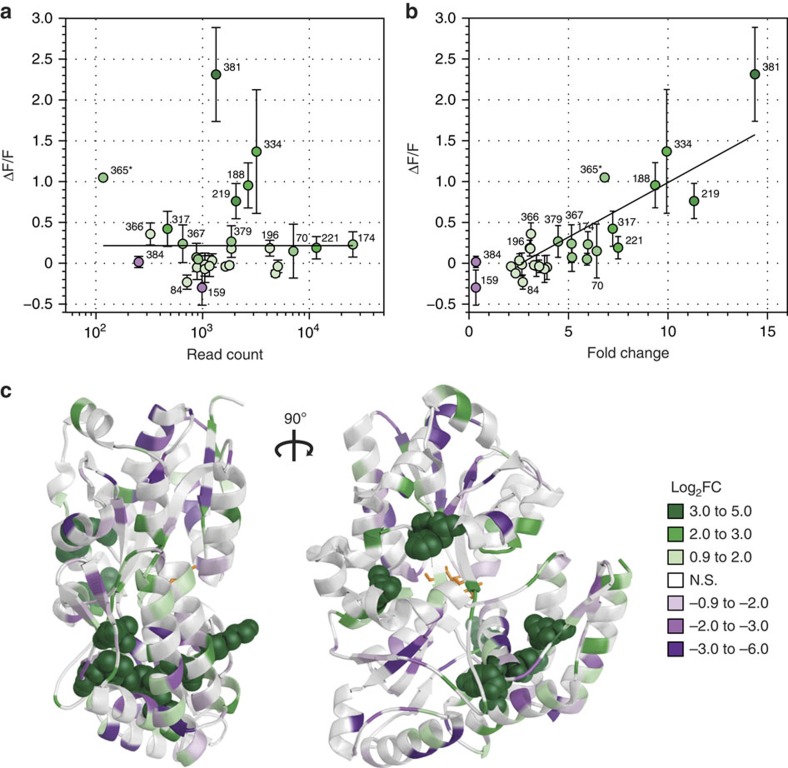
DIP-seq with successive rounds of positive and negative selection enriches for functional biosensors. (**a**) Activity versus final NGS read count. Activity is reported as mean±s.d. of Δ*F*/*F* from at least three biological replicates (asterisk denotes one sample with two replicates). Read count is combined from two biological replicates from the final sort 3 library. Activity is not correlated with final read count (Pearson's *R*^2^=8.6 × 10^−5^, *P*=0.96). (**b**) Activity versus enrichment. Activity is as shown in **a**. Enrichment, shown as fold change, is calculated from two biological replicates comparing the initial and final library NGS read counts. Calculated fold change with *P*<0.1 shown. Activity is linearly correlated with enrichment (Pearson's *R*^2^=0.73, *P*=5.3 × 10^−11^). Colours of points in **a**,**b** are binned based on fold-change values after all sorting rounds. (**c**) Enrichment of cpGFP-insertion sites (log_2_ of fold change) mapped onto TMBP crystal structure (PDB 1EU8). Sites cleared from the library are set at a value of −5. Colours are binned for enrichment values, with white representing sites of nonsignificant enrichment (*P*⩾0.1). Sites in the top bin, having the highest enrichment, are highlighted by sphere representation of their side chains.

Functional assay of the final libraries revealed a strong correlation between enrichment and dynamic range of a biosensor. *In vivo* activity screening of individual clones after the third sort identified several biosensors (cutoff of Δ*F*/*F*⩾0.5) but sequencing revealed only a limited diversity of insertion sites (Supplementary Fig. 6). This observation agreed with NGS data, which confirmed that a small number of constructs dominated the library. As a result, there was no significant correlation between biosensor activity (that is, the Δ*F*/*F* value) and final NGS read count (Fig. 4a). Analysis of construct abundance in the NGS data revealed an ∼1,000-fold variation in the naive library, suggesting initial bias in a library limited the utility of this metric (Supplementary Fig. 7). In contrast, there was a very strong correlation between activity and the fold enrichment of individual biosensors between the final sorted library and the initial naive library (Fig. 4b). Analysis of biosensor activity versus fold-enrichment at individual rounds did not reveal this trend (Supplementary Fig. 8). The correlation only emerged as a result of multiple sorting rounds (Fig. 4b). Thus, our data indicate that successive rounds of profiling with FACS and NGS are crucial to enrich and track functional, in-frame, fluorescent biosensors. Specifically, DIP-seq analysis of the initial and final libraries may be crucial in identifying rare variants that are nonetheless highly functional biosensors.

Mapping of NGS fold-enrichment data (Fig. 4c), as well as biosensor activity (Supplementary Fig. 9 a,b) on the structure of TMBP, revealed that the most enriched—and the most active—sites were three-dimensionally clustered in two distinct regions of the protein. Interestingly, although MBP and TMBP are homologous (28% sequence identity^15^), functional insertion sites for each protein did not overlap substantially (Supplementary Fig. 9c). In addition, some of the best insertion sites from one protein backbone did not produce significant biosensors in the other. These included site 302 in MBP, representing Tre-334 in TMBP (Fig. 2d) and sites 196/197 in TMBP, representing Mal-169/Mal-170 in MBP (Supplementary Fig. 10). These observations further underscore the utility of DIP-seq and the inconsistent linkage between homology and allostery^26^.

### Improving *in vivo* activity and dynamic range of biosensing

To demonstrate the potential of our novel biosensors, one insertion site was optimized for use in physiological experiments. Specifically, Tre-334—a biosensor found in all three replicate libraries—was optimized by varying the amino-acid linkers connecting cpGFP and TMBP (Supplementary Fig. 11a), and subsequently screened for enhanced function. We generated a library of variants containing zero to three amino acid linkers at this insertion site and subjected the library to a single round of FACS screening in the presence of saturating trehalose (Supplementary Fig. 11b). *In vivo* screening of three 96-well plates revealed that the vast majority of variants were functional biosensors and had dynamic ranges greater than the parental biosensor (Supplementary Fig. 11d). This suggests that performing DIP-seq with a fixed linker is sufficient to identify allosteric hotspots within proteins. Then, once hotspots are identified, linker optimization can be used to further increase the dynamic range as needed.

**Figure 5:**
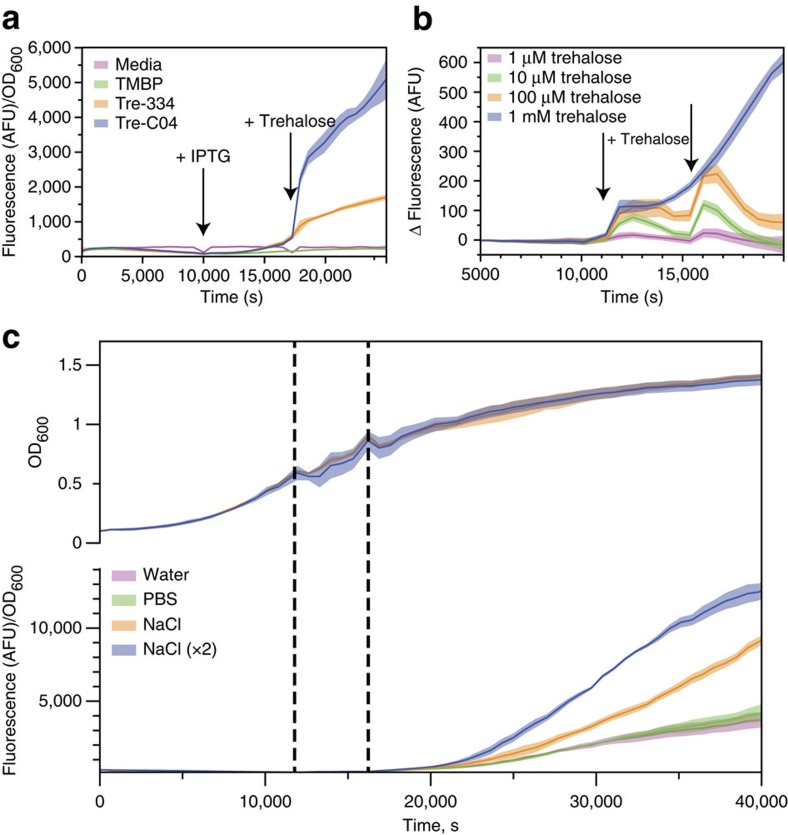
The optimized trehalose biosensor reports trehalose concentrations *in vivo*. (**a**) The fluorescence response of the parental and optimized trehalose biosensors, Tre-334 and Tre-C04, respectively, to 1 mM trehalose addition (indicated by arrow). Negative controls of media and TMBP are also shown. Data are mean±s.d. for three replicates. (**b**) Response of optimized trehalose biosensor, Tre-C04, to 1, 10, 100, or 1000 μM trehalose during exponential growth. Arrows indicate the timing of trehalose addition. Fluorescence was background corrected against a culture treated with water instead of trehalose. Data are mean±s.d. for 12 replicates. (**c**) OD_600_ (upper panel) and fluorescence response (lower panel) of Tre-C04 to the addition of water, PBS or 300 mM NaCl (once or twice). Dashed lines indicate additions. Data are mean±s.d. for four biological replicates.

Tre-C04 was identified in this screen as a high dynamic range biosensor (Δ*F*/*F*=6.3±0.6, s.d.; Fig. 5a and Supplementary Fig. 11c) and chosen for further characterization. Biochemical analysis of purified Tre-C04 confirmed that it was a high-affinity and high-selectivity biosensor with a preference for trehalose (*K*_D_=53±1 nM, s.d.) and lower affinity towards the related maltose (*K*_D_=81±1 nM, s.d.) (Supplementary Fig. 12d). Related sugars displayed little or no activation, even at high concentrations (Supplementary Fig. 12e). The trehalose-activated biosensor was found to be 44% as bright as sfGFP (Methods section) providing further evidence of its utility *in vivo*.

Initial *in vivo* testing verified Tre-C04 responded to trehalose with high activity (Fig. 5a), and so we attempted to determine whether the biosensor was reversible and responded to real-time, dynamic changes. We found that during exponential phase, when *E. coli* do not normally produce trehalose^27^, trehalose addition resulted in an immediate, dose-dependent increase in biosensor fluorescence (Fig. 5b). At intermediate trehalose levels, the fluorescent response eventually diminished with trehalose catabolism and this behaviour repeated on further trehalose additions (Fig. 5b). To demonstrate the linkage between trehalase activity and the observed decrease in fluorescence response with time, cultures were treated with the trehalase inhibitor validamycin A (ref. 28). At all non-saturating concentrations of trehalose tested, validamycin A treatment resulted in a sustained fluorescence response (Supplementary Fig. 13). These experiments confirmed the capacity of our Tre-C04 biosensor to monitor dynamic changes in *in vivo* trehalose levels in real-time and in a dose-dependent manner.

Physiological changes in endogenous *E. coli* trehalose levels can also be monitored with this optimized biosensor. It is known that the trehalose biosynthetic genes are upregulated during the transition to stationary phase^27^. The expression of these genes is also induced to a greater extent during osmotic upshift by the addition of 300 mM sodium chloride during exponential growth^27^,^29^. As expected, Tre-C04 fluorescence levels do not begin rising until the cells enter stationary phase and the addition of sodium chloride potentiates trehalose levels by osmotic upshift (Fig. 5c). In previous reports of stationary phase trehalose accumulation, trehalose levels were undetectable due to limitations in the sensitivity of the thin-layer chromatography assay^29^. Using high-performance anion-exchange chromatography with pulsed amperometric detection (HPAEC-PAD), a significantly more time- and labour-intensive method that does not permit real-time monitoring, we independently measured trehalose levels and found a correlation between Tre-C04 fluorescence signal and intracellular trehalose (Supplementary Fig. 14). Tre-C04, as a real-time biosensor, thus appears to be an extremely sensitive and faithful reporter of trehalose levels.

## Discussion

Here we present DIP-Seq as a robust and generalizable method that couples comprehensive domain-insertion library creation with FACS and NGS to rapidly identify allosteric sites within target protein domains. This method allowed us to create functional, high-dynamic-range biosensors that probed dynamic changes in *in vivo* metabolite concentrations. Collectively, the unbiased and high-throughput nature of DIP-seq sidesteps the difficult task of predicting optimal insertion sites for coupling independent proteins.

Relatedly, DIP-seq provides the ability to elucidate trends that would be invisible to traditional functional screening. Previous studies engineering SFPBs garnered functional data on very few insertion sites because of the laborious nature of their methods^19^. Alternatively, sequencing-based enrichment analysis, such as that previously used in saturation mutagenesis studies, yields a rich landscape of structure–function information^10^,^11^. The low-stringency screening approach of DIP-seq similarly provides a comprehensive view of allosteric hotspots in a binding protein. A high-stringency screen (or continued rounds of screening) would likely enrich a handful of functional biosensors, but the decreased library diversity would also lead to loss of structure–function NGS data. In addition, sequencing-based enrichment analysis is advantageous when the initial abundance of variants is non-uniform. We observe an ∼1,000-fold variation in construct abundance in the initial library pool (Supplementary Fig. 7a). Although this may be specific to our technique, creating a uniform library is a general problem of molecular biology methods^30^. DIP-seq thus generates a broad picture of allosteric coupling regardless of each variant's abundance in a library.

Furthermore, comprehensive mapping of allosteric hotspots may prove helpful in the construction of optimal biosensors. An important consideration is that libraries in this study had fixed inter-domain linker sequences. Subsequent optimization of these sequences in highly enriched variants, a known technique for SFPB improvement^19^,^31^, led to higher dynamic ranges. Further study is therefore needed to determine if less-enriched allosteric hotspots could produce superior biosensors with similar optimization methods. Similarly, while highly enriched members of this fixed-linker library showed robustness to linker sequence diversity (Supplementary Figs 4b and 11d), it is unclear if DIP-seq with a different fixed sequence would result in the same rank order of hotspots. Therefore, the comprehensive dataset DIP-seq provides is a potentially valuable roadmap in constructing the ‘best' biosensor with a given LBD.

As a method for the rapid construction of metabolite biosensors, DIP-seq should prove useful in expanding the palette of SFPBs. This raises the question: can current ligand-binding domains span the space of known metabolites? More than 25,000 bacterial ligand-binding proteins are found in the PFAM database^32^. These include bacterial periplasmic binding proteins from the ATP-binding cassette family of transporters, which are known to bind many diverse ligands^4^,^33^. Given the scalability of DIP-seq, and the potential for computational redesign of ligand-binding proteins^34^, we believe that fluorescent biosensors can readily be fashioned for many molecules of interest.

We show this method can be used to interrogate the periplasmic binding proteins MBP and TMBP for allosteric potential and ultimately, to isolate high-dynamic-range and specific biosensors for the molecules maltose and trehalose. There is no SFPB for trehalose; the biosensor constructed here allows for real-time trehalose detection without the need for lengthy sugar extraction and analysis. Interestingly, despite homology between MBP and TMBP, identified allosteric sites in one homologue are not functional in another (Fig. 2d and Supplementary Fig. 10). This agrees with previous work^26^ and underscores the utility of applying DIP-seq across a family of closely related proteins.

While the work presented here is specifically in the context of SFBPs, DIP-seq may prove useful in probing the general phenomenon of protein allostery. Many forms and mechanisms of protein allostery have been discovered, all of which transmit information from one protein site to another^35^. This field of study has moved from classic, structure-based views of proteins^36^,^37^ to include statistical and thermodynamic perspectives informed by protein dynamics^38^,^39^. This revised view of allostery predicts that most proteins have inherent allosteric potential if correctly coupled and/or perturbed^40^,^41^. Thus, using DIP-seq we can create populations of allosteric couplings between a specific ‘input' and ‘output' domain to investigate the potential for allostery. For example, DIP-seq could be used to query output domains with other functions such as genome editing, like the CRISPR-Cas9 protein, as opposed to the GFP used in this work^42^,^43^.

Overall, DIP-seq allows for the interrogation of protein allostery through the identification of potential hotspots without concern for the underlying mechanism. Using controlled sets of domains with diverse allosteric connections gives insight that extant naturally evolved proteins may not. This high-throughput approach allows for the generation of data that cannot be ascertained from the engineering of a single insertion site or target protein. Ultimately, such data may uncover trends that illuminate and inform the structural and dynamical determinants of protein allostery.

## Methods

### Domains

The DNA sequence of MBP (*malE*), encoding amino acids 1–370 of the mature peptide, not including the signal peptide, was amplified from the *E. coli* genome. A point mutation was used to remove a BsaI recognition site (Gly19, GGT->GGA; see Supplementary Table 1 for ORF sequences). The DNA sequence corresponding to amino acids 46–450 of *T. litoralis* TMBP (*malE*) C-terminally fused to the five N-terminal amino acids of mature MBP^15^ was codon optimized for expression in *E. coli* and synthesized as a gBlocks gene fragment (IDT). Numbering of TMBP amino acids follows Diez *et al*.^15^, where the fusion protein is numbered consecutively 1–410. cpGFP was created from standard EGFP^44^ to match the amino-acid sequence of the cpGFP used in previous maltose biosensor constructs^19^. Briefly, EGFP 147–238 was fused N-terminally to EGFP 1–146 with a GGTGGS linker in between. Mutations made, relative to EGFP, were H148Y, Y151F, V163A, D180Y, T203V, V93I and Y145F.

### Modified transposons

The minimal transposon Entranceposon (M1-CamR) was purchased from Thermo Fisher Scientific (catalogue # F-760). Using F-760 as template, Golden Gate cloning^18^ was used to remove two internal BsmBI restriction sites along with the NotI sites at the transposon ends (returning the ends to the native Mu R1R2 sequences). The F-760 template was amplified in three pieces (see Supplementary Table 1 for primers) and cloned into pET47b (Novagen) using BsaI enzyme (New England Biolabs). This new plasmid was used as template for creating all modified transposons.

Minimal transposons have ends that are exact reverse compliments (R1R2 sequences). Therefore, amplification as two halves (with at least 3% dimethylsulfoxide in PCR reactions) and reassembly into a destination vector was necessary to create modified transposons and to provide a way to create asymmetric modifications. Splitting the CmR resistance gene between the two cloning inserts also allowed for the selection of the correctly assembled transposon. Internal primers, annealing within *CmR*, were combined with R1R2 (ref. 45) primers that introduced modifications (see Supplementary Fig. 1b for modifications and Supplementary Table 1 for primers). The two modified transposon halves were cloned into a pUC19 backbone with BsaI or BsmBI (New England Biolabs) to form a transposon-propagation plasmid. R1R2 primers included restriction sites (BglII and HindIII, Thermo Fisher Scientific) that were used to excise the transposons from transposon-propagation plasmids. This created ‘pre-cut' transposon DNA that, once gel purified from the plasmid backbone, was used in minimal *in vitro* transposition reactions^16^. The transposon used for creating domain-insertion libraries, an asymmetric version of BsaI-1 that includes mutations to the cut sites (Supplementary Fig. 1b,c), was cloned in the same way and named Mu-BsaI. Its transposition efficiency was equivalent to the parent construct, BsaI-1. Primers were designed to amplify cpGFP for Golden Gate cloning into the BsaI sites of Mu-BsaI (Supplementary Table 1).

Transposition efficiency of modified transposons was tested in 10 μl reactions with the following: 2 μl 5 × MuA reaction buffer; 100 ng modified transposon (purified from transposon-propagation plasmid); 100 ng pUC19 plasmid; and 0.5 μl 0.22 μg μl^−1^ MuA transposase enzyme (Thermo Fisher Scientific). Reactions were carried out for 18 h at 30 °C followed by 20 min at 75 °C to heat inactivate MuA transposase. The entire reaction was added to 100 μl XL1-Blue chemically competent cells and transformed by standard heat-shock procedures. Dilutions of the recovered transformation cells were spotted onto lysogeny broth (LB) agar plates with 15 μg ml^−1^ chloramphenicol and 25 μg ml^−1^ carbenicillin. Efficiency was calculated by comparing colony count of modified transposons with the count from unmodified R1R2 transposon.

### Domain-insertion libraries

The ORF of the target gene was cloned into the staging plasmid using BsaI (New England Biolabs). The same 4-basepair cut sites used to clone into the staging plasmid are also flanked on the opposite side by BsmBI recognitions sites, permitting excision of the ORF-transposon library after transposition.

Transposition reactions were a total volume of 20 μl with the following components: 4 μl 5 × MuA reaction buffer; 100 ng Mu-BsaI (purified from transposon-propagation plasmid); staging plasmid DNA (∼0.5 molar ratio to transposon DNA); and 1 μl 0.22 μg μl^−1^ MuA transposase enzyme (Thermo Fisher Scientific). Reactions were incubated for 18 h at 30 °C followed by 10 min at 75 °C to heat inactivate MuA transposase. Completed reactions were cleaned up with a DNA Clean & Concentrator-5 Kit (Zymo Research Corp.), eluting with 6 μl of water. A volume of 2 μl was transformed into 25 μl of electrocompetent 10G *E. coli* cells (Lucigen Corp.) according to the manufacturer's instructions. An aliquot of the recovery culture was spread on an LB agar plate with appropriate antibiotics to assess reaction efficiency. Each transformation produced ∼10^5^ constructs with successful insertions, which covers the number of possible insertion positions in the plasmid (∼6,000) over 15-fold. Cells were then pelleted and resuspended in 50 ml LB with 25 μg ml^−1^ chloramphenicol and 50 μg ml^−1^ carbenicillin to select for transposon insertions and the staging-plasmid backbone, respectively. The culture was grown overnight at 37 °C, 250 r.p.m., and the staging library DNA was collected with a HiSpeed Plasmid Midi Kit (Qiagen).

To isolate ORF DNA that contained a transposon insert, the staging library DNA was treated with BsmBI (New England Biolabs) and size selected on a Tris acetate EDTA (TAE) 1% agarose gel. The band corresponding to the size of the target gene ORF plus a single transposon insertion was gel extracted and the DNA recovered using a Zymoclean Gel DNA Recovery Kit (Zymo Research Corp.). The recovered DNA was then cloned into an expression plasmid (pTKEI-Dest—Supplementary Fig. 15—for MBP and pTKE-Dest for TMBP) using BsmBI enzyme. Briefly, 40 fmoles of extracted ORF-transposon was mixed with 40 fmoles expression plasmid, 10 units BsmBI (NEB), 800 units T4 DNA Ligase (NEB) and 1 × T4 DNA Ligase Reaction Buffer (NEB) in a total volume of 20 μl. The reaction was incubated 2 min at 45 °C, 5 min at 16 °C (first two steps cycled 50 times), 20 min at 60 °C and 20 min at 80 °C. Reactions were purified using a DNA Clean & Concentrator-5 Kit and eluted with 6 μl water. A volume of 2 μl was transformed into 25 μl of electrocompetent 10G *E. coli* cells. An aliquot of the recovery culture was spread on an LB agar plate containing 5-bromo-4-chloro-3-indoyl-β-D-galactopyranoside/isopropyl β-D-1-thiogalactopyranoside (X-gal/IPTG) to assess reaction efficiency. Plasmids still containing a *lacZ* drop-out cassette made up <0.1% of the population for every sample. Cells were then pelleted and resuspended in 6 ml LB with 1% glucose and 25 μg ml^−1^ chloramphenicol. The culture was grown overnight at 37 °C, 250 r.p.m., and the transposon-insertion library DNA was collected with a QIAprep Spin Miniprep Kit (Qiagen).

Golden Gate cloning was used to exchange the modified transposon cassette with the PCR-amplified cpGFP to create the final domain-insertion library. Briefly, 40 fmoles of each DNA component was mixed with 10 units BsaI (NEB), 800 units T4 DNA Ligase (NEB) and 1 × T4 DNA Ligase Reaction Buffer (NEB) in a total volume of 20 μl. The reaction was incubated 2 min at 37 °C, 5 min at 16 °C (first two steps cycled 50 times), 20 min at 60 °C and 20 min at 80 °C. Reactions were purified using a DNA Clean & Concentrator-5 Kit and eluted with 6 μl water. A volume of 5 μl was transformed into 50 μl of electrocompetent Tuner cells (for TMBP libraries, also containing MiniF *lacI*^*q*^ isolated from NEB Express *I*^*q*^ cells). An aliquot of the recovery culture was spread on an LB agar plate containing 25 μg ml^−1^ chloramphenicol to assess reaction efficiency. Cells were then pelleted and resuspended in 6 ml LB supplemented with 1% glucose and 60 μg ml^−1^ kanamycin. The culture was grown overnight at 37 °C, 250 r.p.m., and used the next day to inoculate samples for FACS. A glycerol freezer stock was also made, and the remaining culture used to extract the library DNA (for use in NGS) with a QIAprep Spin Miniprep Kit (Qiagen).

Two MBP-cpGFP libraries were created with independent transposition and cloning steps. Three TMBP-cpGFP libraries were created with independent transposition and cloning steps. Each library replicate is named with a consecutive numbering system, for example, TMBP libraries 1, 2 and 3.

### Fluorescence-activated cell sorting

A 100 μl aliquot of the overnight library culture was used to inoculate 5 ml of MOPS EZ Rich Defined Medium (Teknova) containing 60 μg ml^−1^ kanamycin and 0.4% glycerol as a carbon source instead of glucose. The culture was grown at 37 °C, 250 r.p.m. until the OD_600_ was ∼0.6 and then protein expression induced with IPTG (0.5 mM, Teknova). After 4 h, an aliquot from each library was moved to a new tube where ligand was added to a final concentration of 1 mM, either trehalose (Sigma-Aldrich) or maltose (Fisher Chemical). All samples were incubated at 37 ^°^C, 250 r.p.m. for a further 30 min before FACS. Just before FACS, the samples were diluted 1:100 in phosphate-buffered saline (PBS) or 1:100 in PBS with 1 mM ligand and kept on ice. FACS was performed using a Sony SH800 cell sorter (Sony Biotechnology) equipped with a 488-nm excitation laser and an FL1 (525/50 nm) emission filter. In all, 25,000 events of each sample were recorded for analysis. Expressed ligand-binding protein (MBP or TMBP) was used as a negative control to establish a lower boundary of the gate used for sorting fluorescent events. The same instrument and procedure was used to make single-cell measurements of biosensors with and without ligand.

Cells were sorted into 5 ml LB medium supplemented with 1% glucose. Immediately after sorting, samples were incubated at 37 °C, 250 r.p.m. for 1 h. Following this, the volume was adjusted to 15 ml with LB media and 1% glucose. A volume of 100 μl of a 1:50 dilution of the cells in the same media was plated on to LB agar plates supplemented with either 60 μg ml^−1^ kanamycin or (for MiniF plasmid containing cells) both 30 μg ml^−1^ kanamycin and 12.5 μg ml^−1^ chloramphenicol. The remaining, recovered cell cultures were supplemented with 60 μg ml^−1^ kanamycin and incubated at 30 °C, 250 r.p.m. overnight. If samples were to be sorted again, overnight cultures were used to inoculate new FACS samples, repeating the steps above. A glycerol freezer stock was made from the overnight growth and the remaining culture used to extract library DNA (for use in NGS) with a QIAprep Spin Miniprep Kit (Qiagen).

For the two MBP-cpGFP libraries, each naive pool was treated and sorted with 1 mM maltose. An initial filtering sort was used on ‘yield' sorting mode of the instrument and sorted cells were collected in a fresh tube with no medium. This gate was set at the low-fluorescence threshold, collecting above ∼550 arbitrary fluorescence units (AFU). The collected cells were put through the sorter a second time on ‘purity' sorting mode. The gate was set just above the low-fluorescence threshold, collecting above ∼850 AFU. Approximately 1.5 × 10^5^ cells were collected from each library.

Three FACS screens were carried out on TMBP domain-insertion libraries. For each of the three naive TMBP-cpGFP insertion libraries, expressed libraries were treated with 1 mM trehalose, and 10^6^ fluorescent events above the low-fluorescence threshold (∼550 AFU) were sorted. This sort was defined as sort 1. For the second round of sorting (that is, sort 2), the recovered libraries were processed as described for the initial, naive libraries except that the libraries were not treated or sorted with trehalose. The gate used for these sorts was ∼300–1,100 AFU. In the final round of sorting (sort 3), the expressed libraries were again treated and sorted in the presence of 1 mM trehalose. The gate used was ⩾1,050 AFU.

### Screening for biosensors

A 96-well plate of colonies from plated cells on LB agar was picked into 200 μl PBS. No cells, MBP/TMBP, and cpGFP controls were always included. A volume of 5 μl of cell-PBS mix was used to inoculate 200 μl of LB media containing 10% glycerol, 1% glucose and 60 μg ml^−1^ kanamycin. Plates were incubated at 30 °C, 750 r.p.m. overnight and the following day 5 μl of overnight culture was used to inoculate 200 μl MOPS EZ Rich Defined Medium (Teknova) containing 60 μg ml^−1^ kanamycin and 0.4% glycerol as a carbon source instead of glucose. The overnight starter-culture plates were frozen at −80 °C for storage. The freshly inoculated samples in a 96-well plate (Costar 3631 with an attached low evaporation lid, Costar 3370) were incubated in an Infinite M1000Pro plate reader (Tecan) at 37 °C and the OD_600_ and fluorescence (485/5 nm excitation and 515/5 nm emission) monitored. For OD_600_, 25 flashes were taken. For the fluorescence, the plate was read from the bottom, the gain manually set to 80 and 50 flashes in mode 1 (400 Hz) were taken. After a complete cycle of measuring the OD_600_ and fluorescence, the plates were shaken for 600 s in double orbital mode with amplitude of 1 mm (frequency 306 r.p.m.) before measurements were again taken. Approximately 2.5 h after starting the measurements, the programme was paused and IPTG added to a final concentration of 0.5 mM. The programme was restarted and allowed to continue for an additional 2 h. After 2 h, the programme was again paused and ligand (maltose or trehalose) in PBS was added to a final concentration of 1 mM or PBS alone was added. The programme was restarted and allowed to continue for at least a further 1.5 h. The data from the plate reader were subsequently analysed using MATLAB (Mathworks). To normalize the data for differences in cell growth, the fluorescence at each time point was divided by the OD_600_ at the same time point. The normalized fluorescence values for the time point immediately before ligand addition was used as the pre-ligand fluorescence. For the post-ligand fluorescence, the normalized fluorescence from time points 2–6 immediately after ligand addition were averaged and used. The Δ*F*/*F* (Δ*F*/*F*=(*F*_ligand_–*F*_0_)/*F*_0_) was calculated and normalized to the control protein. The insertion site of a construct was determined by isolation of the plasmid DNA and sequencing (Quintara Biosciences).

For each sorted MBP-cpGFP library, two 96-well plates were tested for biosensor activity. One plate of sorted samples from library 2 had every construct sequenced. The other three plates tested had constructs sequenced if they were found to have an activity measurement of Δ*F*/*F*>0.5.

For each of the three TMBP-cpGFP libraries, after sort 3, a 96-well plate of samples was tested for biosensor activity. The top five wells based on activity values (Δ*F*/*F*) were sent for sequencing. To identify the insertion sites for a complete 96-well plate of samples, an aliquot of the glycerol stock from library 1 after sort 3 was diluted 1:10^5^ in LB media and plated on to LB agar plates supplemented with 60 μg ml^−1^ kanamycin. The DNA from a 96-well plate of colonies was isolated and sequenced. To match the insertion site with biosensor activity, the normalized Δ*F*/*F* was calculated from three replicates (except for Tre-217, which was in duplicate), as described above.

To evaluate linear correlations between activity and either enrichment or read count, linear least squares regression was performed using the SciPy Python package. A two-sided *P* value is reported where the null hypothesis is that the slope is zero. Data sets and calculated fit lines were plotted using DataGraph (Visual Data Tools).

### Biosensor linker optimization

The Mal-170 construct was tested by varying linkers between MBP and cpGFP. The forward primer set cpEGFP-MBP170-F0,1,2,3,4 and reverse primer set cpEGFP-MBP170-R0,1,2,3,4 (Supplementary Table 1) amplified cpGFP adding zero to four VST codons on either end. Primers were mixed in equal ratios and used in a standard Phusion PCR reaction (New England Biolabs). A pET T7-based expression plasmid (Novagen) containing MBP was amplified with MBP-170-F and MBP-170-R. The purified PCR fragments were cloned together with a BsaI Golden Gate reaction. The completed reaction was purified and concentrated with a PCR clean-up kit, and eluted with 6 μl water. A volume of 2 μl of cleaned-up reaction was transformed into 25 μl of electrocompetent BL21 (DE3) *E. coli* cells. An aliquot of the recovery culture was spread on LB agar plates with 50 μg ml^−1^ carbenicillin and 1% w/v glucose. Colonies were picked for inoculation of two 96-well plates, which were screened for biosensor activity as described above. A range of activity was seen (Supplementary Fig. 4b); the top five constructs were sent for sequencing. This identified constructs Mal-F1 and Mal-B2. These constructs were cloned into pTKEI (Trc-promoter plasmid) for further testing (Supplementary Fig. 4c,d) because we found that biosensor expression from T7-based plasmids showed attenuated activity.

For Tre-334 optimization, the forward primer set Tre-333/334-F0,1,2,3 and reverse primer set Tre-333/334-R0,1,2,3 (Supplementary Table 1) were designed to introduce zero to three degenerate VVC codons on both the 5′- and 3′-ends of the amplified cpGFP template. Each primer was prepared at 25 μM then pooled as forward and reverse primers in the ratio 3^0^:3^1^:3^2^:3^3^ (0:1:2:3 linker codons) and used in a standard Phusion PCR with cpGFP as the template. To amplify the TMBP backbone, primers Tre-G334-F and Tre-P333-R were used in a standard Phusion PCR with TMBP in plasmid pTKE-Dest. Each of the PCR products was electrophoresed on a TAE 1% agarose gel and extracted. The purified products were used in a Golden Gate cloning reaction to construct the TMBP-cpGFP (0–3 VVC) linker library. The products of the Golden Gate reaction were purified using a Clean and Concentrator kit (Zymo) and eluted with 6 μl water.

The purified libraries were transformed with electrocompetent Tuner-MiniF *lacI*^*q*^ cells as described for the domain-insertion TMBP-cpGFP libraries. Expression and sorting of the library was also carried out as detailed for the domain-insertion libraries except that only a single round of sorting in the presence of 1 mM trehalose was performed. The gate used for sorting was a high-fluorescence gate (⩾3,750 AFU).

To determine the range in Δ*F*/*F* for the linker library, three 96-well plates of colonies were screened as described for the domain-insertion libraries. Sequencing of the biosensors with the highest Δ*F*/*F* values identified biosensor Tre-C04, which was used for further characterization.

Biosensors Tre-334 and Tre-C04 along with the controls TMBP and cpGFP were cloned into the expression plasmid pTKEI-Dest (Supplementary Fig. 15) for all further characterization. Unlike pTKE, pTKEI constitutively expresses the *lacI* gene to repress expression of target proteins prior to induction. All expression with these plasmids was carried out in Tuner cells (Novagen).

### Tre-C04 switching during exponential growth

Colonies of Tuner cells (Novagen) harbouring Tre-C04 in pTKEI were grown overnight in LB media supplemented with 1% glucose and 60 μg ml^−1^ kanamycin. The following day, 5 μl of the overnight culture was used to inoculate 200 μl MOPS EZ Rich Defined Medium (Teknova) containing 60 μg ml^−1^ kanamycin and 0.4% glycerol as a carbon source instead of glucose. Samples were grown and monitored as previously described for the *in vivo* screening of the trehalose biosensors except that 2 h after IPTG induction, the 96-well plate was removed and 5 μl of the culture used to inoculate 200 μl of fresh MOPS EZ Rich Defined Medium (Teknova) containing 60 μg ml^−1^ kanamycin, 0.4% glycerol as carbon source and 0.5 mM IPTG. Approximately 3–3.5 h after back-dilution and return to the plate reader, the programme was paused and either water or trehalose at final concentration of 1, 10, 100, or 1000 μM was added. Cultures were again returned to the plate reader and monitored for ∼1 h before another aliquot of water or trehalose was added. The difference in fluorescence between cultures in which trehalose was added versus water was calculated and plotted using DataGraph (Visual Data Tools). The experiment was replicated 12 times.

### Tre-C04 response to validamycin A

To monitor the response of Tre-C04 to validamycin A treatment, Tuner cells (Novagen) harbouring Tre-C04 in pTKEI were treated as described above for Tre-C04 switching during exponential growth except that the first addition of trehalose or trehalose supplemented with validamycin A (50 μM final concentration, Research Products International Corp.) was ∼1.75 h after back-dilution and the second addition was 1.5 h after the first. For samples with zero trehalose, water was added.

### Monitoring *in vivo* trehalose levels during stationary phase

To monitor the *in vivo* accumulation of trehalose in *E. coli*, Tuner cells expressing Tre-C04 were grown and monitored as described for the *in vivo* switching experiment except that instead of trehalose addition, water, PBS or 300 mM NaCl was added. For NaCl, either one or two aliquots was added. For cultures in which only a single addition was made, water was added at the second addition point. The mean and s.d. for the changes in OD_600_ and normalized fluorescence for four samples was determined.

### Extraction and measurement of free sugars

The *in vivo* accumulation procedure was replicated using 50 ml cultures to provide enough cell mass for sugar extraction. Cultures were grown at 37 °C, 250 r.p.m. and sampled periodically. The OD_600_ and fluorescence were measured with an Infinite M1000Pro plate reader using the parameters described above for the *in vivo* screening of trehalose biosensors. On the basis of OD_600_ measurements, 5 × 10^8^ cells were removed from the 50 ml culture, pelleted at 5,000*g* and washed with MOPS EZ Rich Defined Medium containing no carbon source. Cells were treated with 1 ml acetonitrile:methanol:water (40:40:20 by volume). The extraction solution was spiked with 1 μg of arabinose, for use as an internal control, and incubated for 15 min at 50 °C, 1,400 r.p.m. Samples were centrifuged at 20,000*g* for 10 min. The supernatant was removed and stored at −80 °C until HPAEC-PAD analysis.

Before HPAEC-PAD analysis, extracts were thawed and then dried at 50 °C under nitrogen gas. Dried residue was resuspended in 1 ml water and then 500 μl chloroform added. Samples were vortexed and centrifuged at 20,000*g*. The aqueous phase was recovered and analysed by HPAEC-PAD. Briefly, a Dionex ICS-5000 system with a CarboPac PA20 analytical column (3 × 150 mm) and CarboPac PA20 guard column (3 × 30 mm) was used with an isocratic 50 mM NaOH eluent and 0.4 ml ml^−1^ flow rate. Injections were 25 μl per sample. Chromeleon 7 software (Dionex) was used for instrument control, data collection and data analysis. The retention times and calibration curves for maltose, trehalose and arabinose concentrations were determined using pure standards (Sigma-Aldrich).

### *In vitro* biochemistry

A single colony of each of the biosensor and control proteins was transformed into Tuner cells (Novagen) and used to inoculate 10 ml of LB media supplemented with 1% glucose and 60 μg ml^−1^ kanamycin. Cultures were incubated overnight at 30 °C, 250 r.p.m. and then following day used to inoculate 1 l of Autoinduction media^46^. Cultures were incubated for 20–24 h at 22 °C, 250 r.p.m. before collecting at 4,000*g* using a Sorval Legend XTR centrifuge. Cell pellets were resuspended in 30 ml lysis buffer (50 mM sodium phosphate, 300 mM NaCl and 10 mM imidazole, pH7.4) and frozen at −80 °C. Phenylmethylsulfonyl fluoride and DNase I were added to the thawed cell pellets at final concentrations of 1 mM and 10 μg ml^−1^, respectively. Cells were lysed with an EmulsiFlex C3 (Avestin) at 20,000 psi for three passages. Lysate was centrifuged at 18,000 r.p.m. for 30 min using a JA-20 fixed angle rotor (Beckman Coulter). The clarified lysate was purified using Ni-NTA agarose (Qiagen). The purified proteins were concentrated and exchanged into PBS buffer using a 10,000 Da NMWO Vivaspin Turbo 15 Centrifugal Concentrator (Sartorius). To verify purity, aliquots of purified proteins were analysed by SDS–PAGE.

For MBP-cpGFP Mal-B2, the protein was diluted to ∼10 μM in PBS. In a standard 384-well plate, 20 μl of 2 × ligand (in PBS) was added to 20 μl of protein. No-ligand measurements were 20 μl PBS added to 20 μl of protein. Excitation/emission (485/515 nm) was measured on an Infinite M1000Pro (Tecan) plate reader after PBS (with or without ligand) addition. Samples were measured in triplicate, and the mean and the s.d. of the Δ*F*/*F* values determined. Plots were generated using Prism (GraphPad Software).

For TMBP-cpGFP biosensors, fluorescence images of the purified proteins were obtained by adding either PBS or trehalose at a final concentration of 1 mM to 1 μM protein. Samples were imaged using a ChemiDoc MP (Bio-Rad) using the epi-blue LED module.

To determine Tre-C04's fluorescence response to sugars, 500 nM of purified protein was treated with PBS or 50 μM glucose, maltose or trehalose. Using an Infinite M1000Pro (Tecan) plate reader, samples were excited at 485/5 nm, and a fluorescence scan was taken from 500 to 550 nm (5 nm bandwidth). Samples were measured in triplicate, and the mean and the s.d. determined. Plots were generated using Prism (GraphPad).

To obtain a dose–response curve, 500 nM purified Tre-C04 biosensor was treated with 0–50 μM glucose, maltose or trehalose. To assess Tre-C04's response to other possible ligands, the protein was incubated with 1 mM sugar substrates. Using an Infinite M1000Pro (Tecan) plate reader, samples were excited at 485/5 nm and emission measured at 515/5 nm. Samples were measured in triplicate, and the mean and the s.d. determined. Plots were generated using Prism (GraphPad Software), and the fit of log([sugar]) versus response using four parameters are displayed with the dose–response curves. Under these measurement conditions, it was found that purified Tre-C04 saturated with trehalose was 44% as bright as purified sfGFP.

### NGS and analysis

Library plasmid DNA was sheared with a Covaris S220 focused-ultrasonicator using AFA microTUBEs (#520052, Covaris) to an approximate size of 250 basepairs. All DNA clean ups of NGS samples were done with Agencourt AMPure XP SPRI beads (Beckman Coulter). MiSeq-compatible fragments were prepared from sheared DNA with a NEBNext DNA Library Prep Reagent Set for Illumina (New England Biolabs) and TruSeq DNA Adaptors (#15025064, Illumina). Sheared DNA and prepared samples were analysed for size distribution on an Agilent 2100 Bioanalyzer using DNA 1000 chips (Agilent Technologies). Double-stranded DNA concentrations of the adaptor-prepared samples were measure with a dsDNA BR Assay Kit on a Qubit Fluorometer (Life Technologies). A normalized pool of samples was run on a MiSeq instrument using a MiSeq Reagent Kit v3 (150 cycles, Illumina).

NGS data was analysed with a custom Python pipeline (available at http://github.com/SavageLab/dipseq). Briefly, an initial filter was used to remove reads that did not contain sequence from both the LDB (either MBP or TMBP) and the inserted domain (cpGFP). The remaining reads were stripped of cpGFP sequence and aligned to the LBD ORF. This alignment determined the insertion site of the cpGFP into the LBD ORF for the given read. Because transposition creates duplicated DNA sequences, the DNA insertion site was defined as the last basepair of the LBD (in the direction of transcription) after which the cpGFP was detected. The cpGFP insertion was designated as forward or reverse based on if its ORF is in the same or opposite direction of the LBD, respectively. The amino-acid insertion site (for forward, in-frame insertions) is then assigned to the codon in which the DNA insertion occurred. If an insert created a forward, in-frame construct, it was categorized as productive. If an insert was out of frame or in the reverse direction, it was categorized as unproductive. The DNA insertion site designation above was used for calculating read counts whether the read detected the N- or C-terminal end of the cpGFP domain. When making enrichment calculations, though, reads identifying N- and C-terminal ends of cpGFP for the same insertion site were used as internal technical replicates. Enrichment values for each insertion site (fold change or log_2_ fold change) were calculated using the DESeq package and *P* values were adjusted for multiple testing using the Benjamini–Hochberg procedure^47^.

For MBP-cpGFP, library 2 was sequenced before and after FACS screening. This resulted in two technical replicates for those two samples when calculating enrichment. For TMBP-cpGFP, two of the three libraries (libraries 1 and 2) were sequenced before and after each sort. Each sample was also sequenced twice on separate MiSeq runs. This resulted in two biological replicates, with four technical replicates each, that were used to calculate enrichment over each FACS screen, and over all three FACS screens (Supplementary Fig. 7b uses enrichment calculated from technical replicates of library 2). For enrichment relative only to productive constructs, the same TMBP-cpGFP data sets were stripped of unproductive insertions and rerun with DESeq to produce new fold changes and *P* values.

### Data availability

Domain-insertion-profile sequencing data that support the findings of this study have been deposited into the NCBI Sequence Read Archive (SRA) with the accession code SRP076284. Sequencing data were analysed with a custom Python pipeline available at http://github.com/SavageLab/dipseq. Processed sequencing data are included in Supplementary Information (Supplementary Data 1 and 2). Plasmids described in this work are available from Addgene. All other data that support the findings of this study are available from the authors on request.

## Additional information

**How to cite this article:** Nadler, D. C. *et al*. Rapid construction of metabolite biosensors using domain-insertion profiling. *Nat. Commun.* 7:12266 doi: 10.1038/ncomms12266 (2016).

## Supplementary Material

Supplementary Information Supplementary Figures 1-15, Supplementary Table 1

Supplementary Data 1 Read count data from MBP-cpGFP and TMBP-cpGFP libraries

Supplementary Data 2 Calculated enrichment data from MBP and TMBP DIP-seq
