# Supplementary material for: Rapid construction of metabolite biosensors using domain-insertion profiling
Source: Nat Commun. 2016 Jul 29;7:12266. doi: 10.1038/ncomms12266 (PMC4974565; doi:10.1038/ncomms12266)
Supplement: Supplementary Information — Supplementary Figures 1-15, Supplementary Table 1 [file ncomms12266-s1.pdf]

Supplementary Information

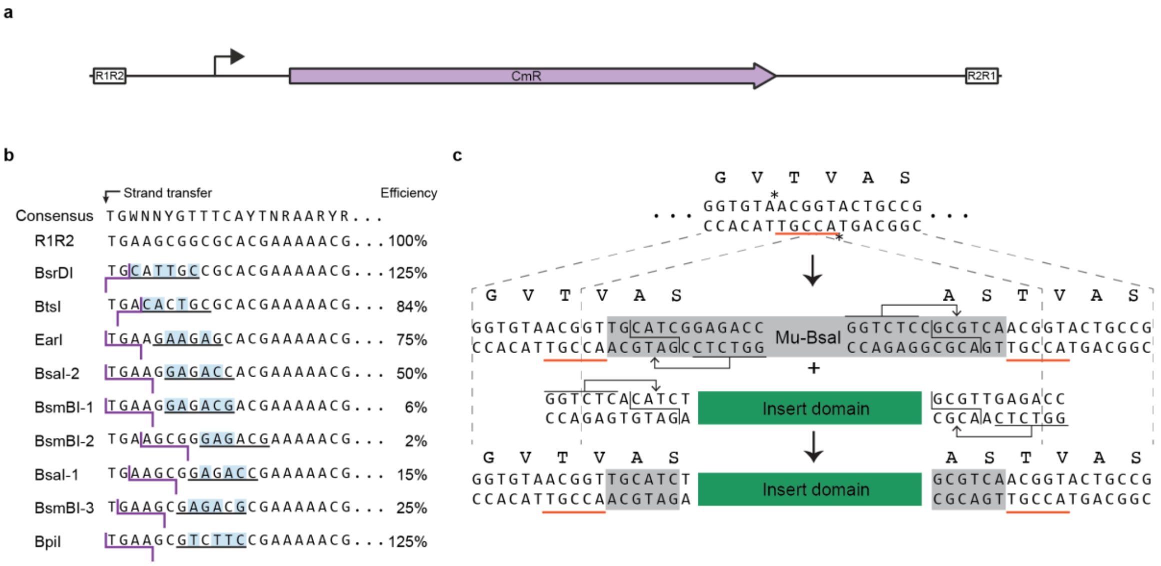

Supplementary Figure 1: Modified transposons for the creation of domain-insertion libraries. (**a**) Schematic of a representative transposon used for *in vitro* DNA insertion. The antibiotic resistance gene (CmR) is shown in purple, with its promoter upstream. R1 and R2 MuA-binding sites are encoded at each end (exact reverse complement of each other). (**b**) Transposition efficiency of modified transposons. Alignment of modified transposon ends, shown 5′ to 3′, with Mu genome consensus and original R1R2 end shown at the top for reference. Each modified transposon is labeled with the restriction site that is created. Mutations relative to original R1R2 ends are highlighted in blue. Restriction site recognition sites are underlined, while cut sites are shown in purple. Note that all restriction sites created are for type-IIs enzymes so sequences in the cut site can be varied at both ends of the transposon to encode for desired amino acids while maintaining the cloning site. (**c**) Schematic of cloning with the modified transposon, Mu-BsaI, to create domain-insertion libraries. An example target sequence is shown at the top, with encoded amino acids shown above. The sites of an example transposon strand transfer are marked with asterisks. Insertion of Mu-BsaI transposon creates a 5 bp duplication of the target sequence (underlined in orange). The insert domain has compatible flanking restriction sites. An additional base (T in this case) must be added in front of the insert domain to keep it in-frame. Colors of the final assembly at the bottom highlight the source of DNA components. The encoded amino acids VAS and AST are considered “linkers” because they are additional relative to the original target and inserting-domain amino acid sequence.

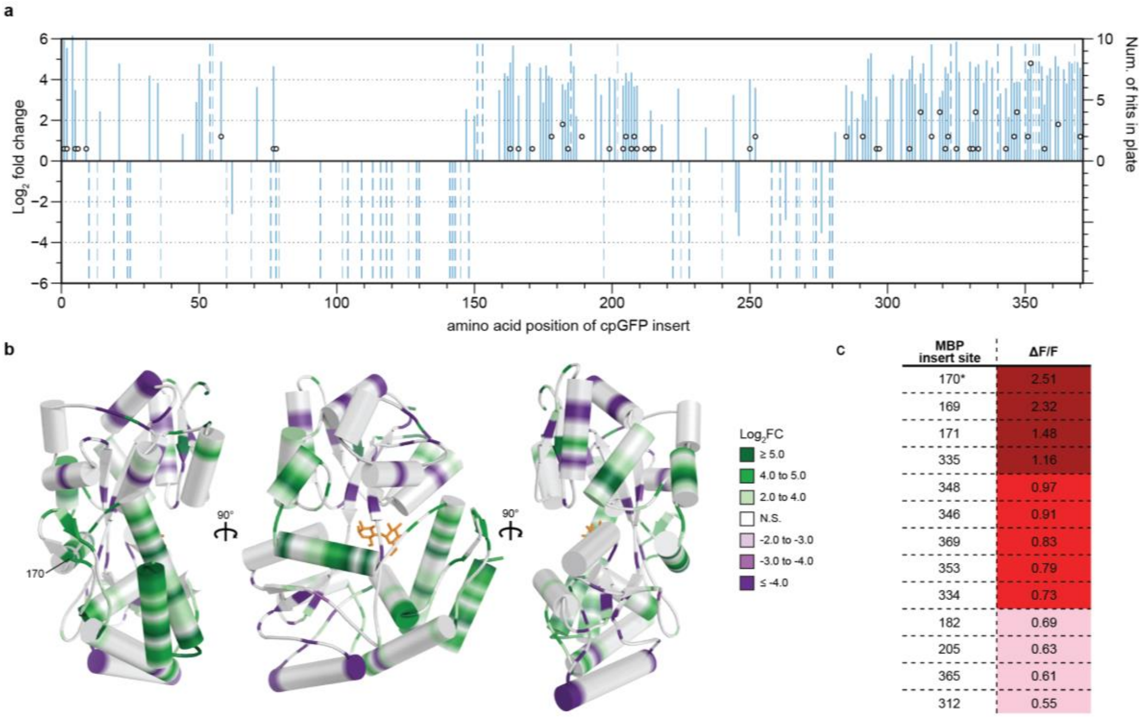

c

MBP insert site	ΔF/F
170*	2.51
169	2.32
171	1.48
335	1.16
348	0.97
346	0.91
369	0.83
353	0.79
334	0.73
182	0.69
205	0.63
365	0.61
312	0.55

Supplementary Figure 2: Analysis of MBP library enrichment and sample activity. (**a**) A profile of enrichment values (*i.e.* $\log_2$ of fold change) for in-frame insertions along the primary sequence of MBP. Enrichment calculated with DESeq comparing post-sort NGS read counts to those from pre-sort library. Calculated enrichment with $P < 0.1$ shown. Sites that went from undetectable (zero reads) to detectable are represented with dashed lines set to a value of 6. Sites that were depleted post-sort are represented with dashed lines set to -6. Overlaid black circles show the number of hits from a sequenced 96-well plate of random post-sort library members (right side of y-axis). (**b**) Enrichment mapped onto MBP crystal structure (PDB 1ANF). Cartoon representation of Figure 2b. Bound maltose is shown in blue. Amino acid 170 indicated with arrows. (**c**) Table of activity values from functional MBP-cpGFP constructs found in plate assays (except site 170, marked with an asterisk, which was constructed afterwards). Activity values are the mean of at least two biological replicates. Highlight colors are the same as in Figure 2d.

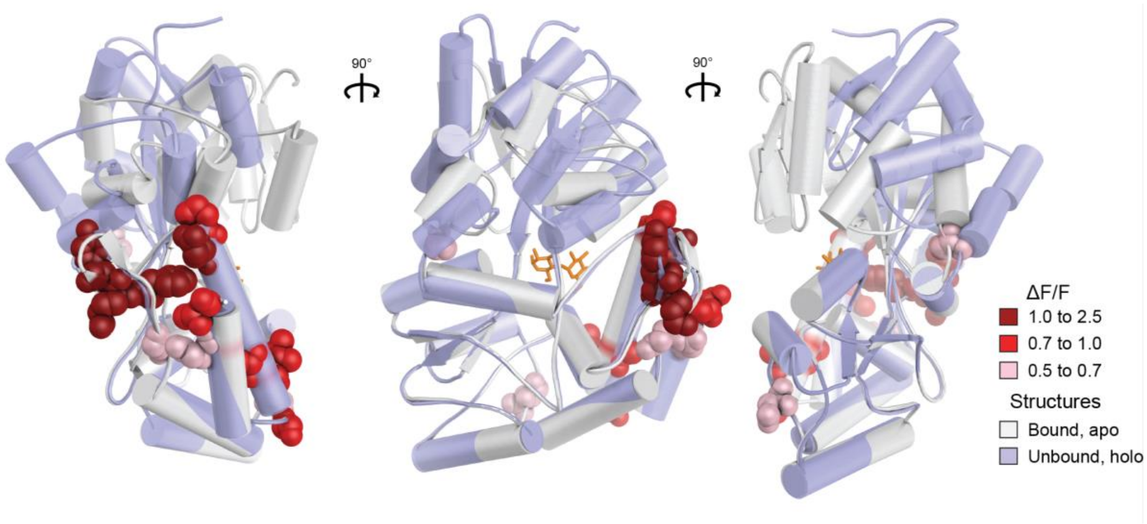

Supplementary Figure 3: MBP functional insertion sites shown relative to bound and unbound structures. Maltose-bound MBP (1ANF, gray structure) shown with functional insertion sites highlighted (from Figure 2c). Unbound MBP (1OMP, light blue structure) is structurally aligned to the bound form. Alignment was carried out with the align function of PyMOL using backbone atoms of amino acids in the C-terminal lobe of the protein (amino acids 114-165, 185-257, 334-370).

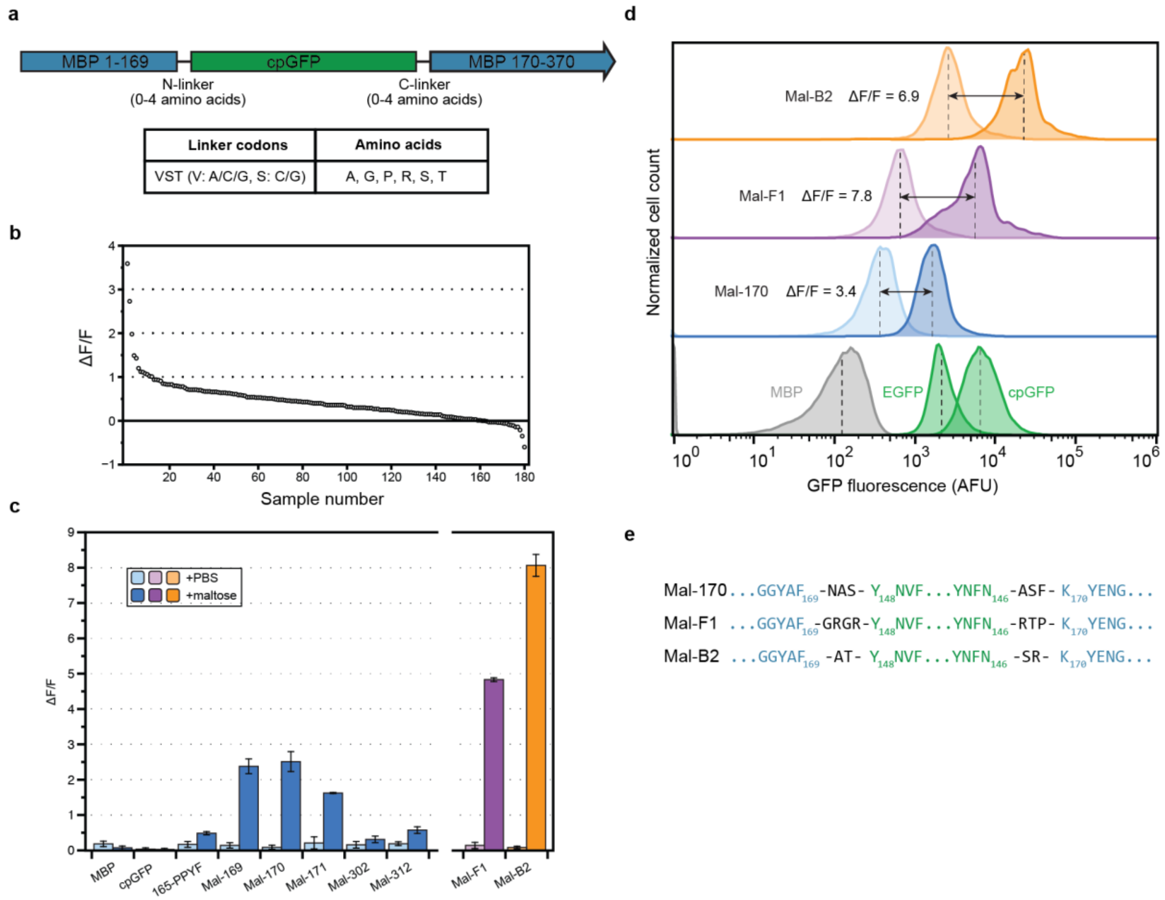

Supplementary Figure 4: Linker optimization of Mal-170. (**a**) Schematic of the construct used for linker optimization. The N- and C-linkers vary from 0 to 4 amino acids, with each having equal probability of Ala, Arg, Gly, Pro, Ser, and Thr (VST codon). (**b**) Representative plate assay activity data of linker library (from naïve library – never sorted). (**c**) Activity measurements of top two hits with replicates (Mal-F1 and Mal-B2) compared to the best constructs from the original library. Values of ΔF/F reported as mean ± s.d. for three replicates. (**d**) Single cell measurements of best hits. Flow cytometry measurements of fluorescence either with (darker color) or without (lighter color) saturating maltose (1 mM), 20,000 measurements for each sample. Dashed lines represent the median of each sample. ΔF/F values are reported next to biosensors, calculated from the median of each sample. (**e**) Linker amino acid sequences of the optimized biosensors, compared to Mal-170.

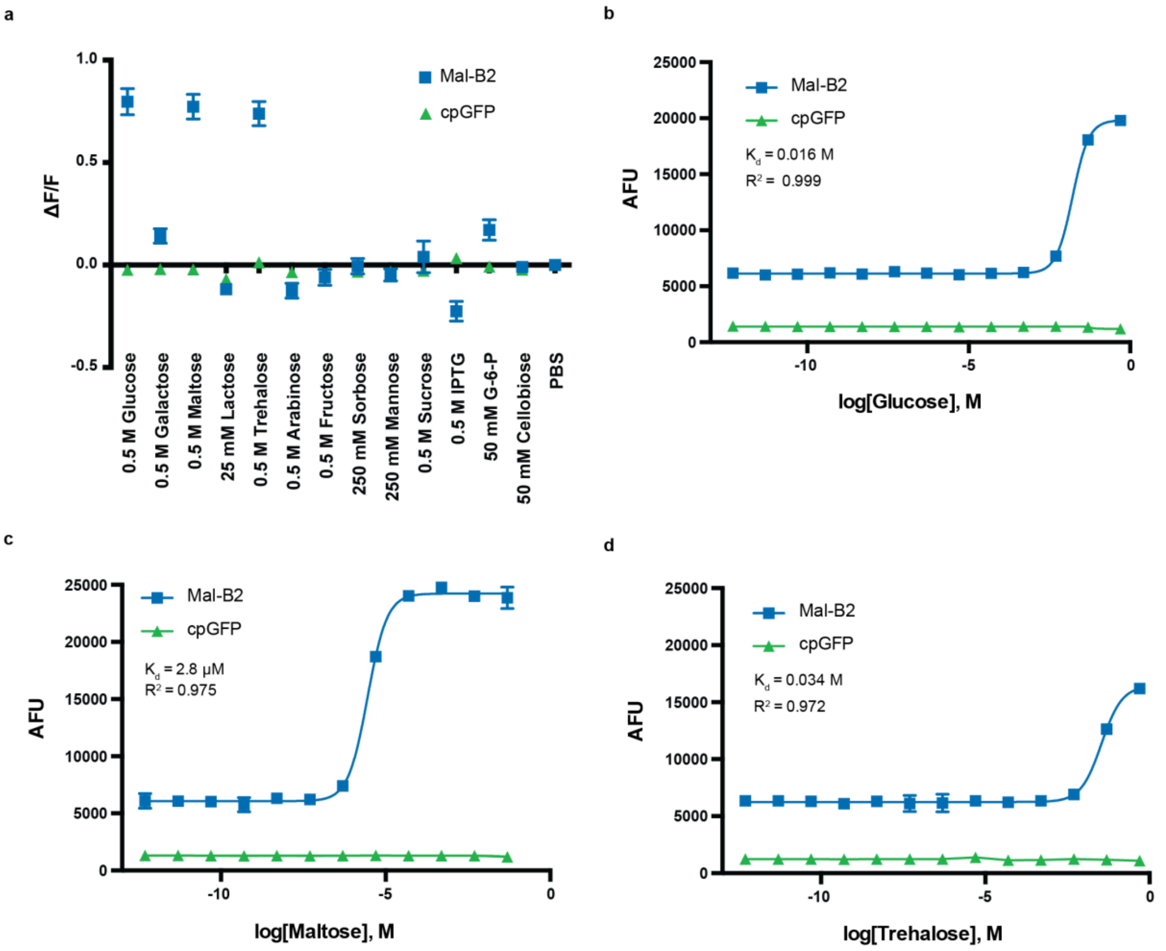

Supplementary Figure 5: *In vitro* analysis of the best optimized MBP-cpGFP biosensor, Mal-B2. (**a**) Activity (ΔF/F) measurements with saturating sugars, as indicated. (**b-d**) Binding curves and K_D calculations for Mal-B2 with (**b**) glucose, (**c**) maltose, and (**d**) trehalose, the substrates showing the highest responses in (a). Data are mean ± s.d. for three replicates.

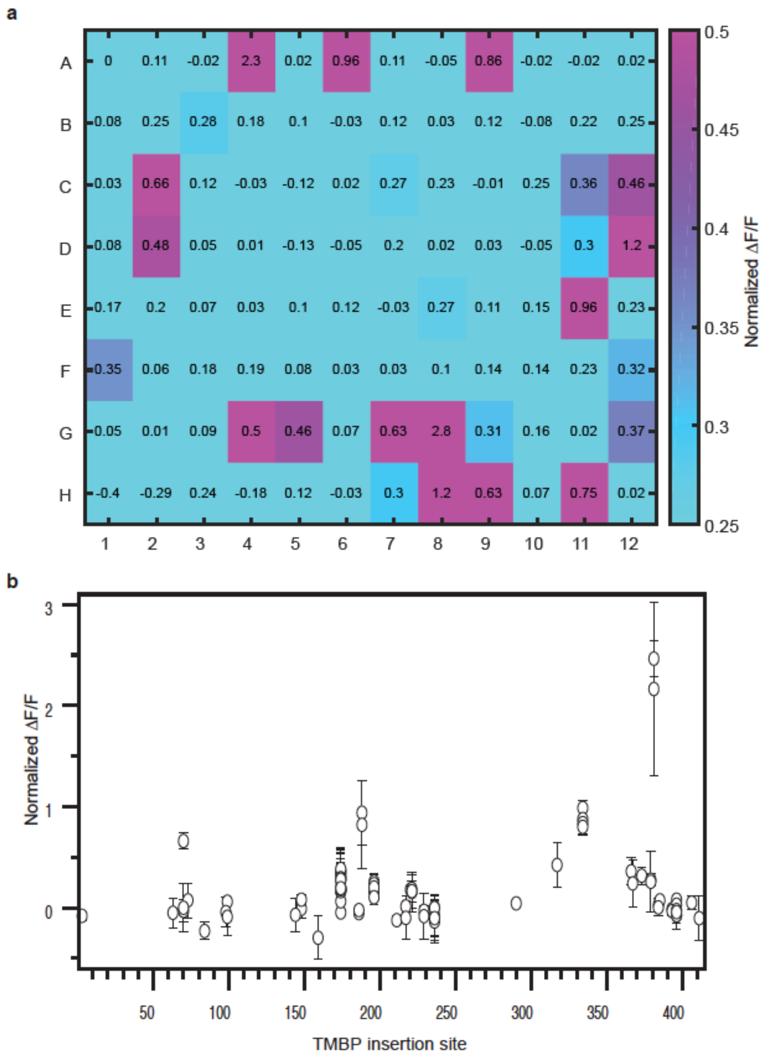

Supplementary Figure 6: DIP enriches for functional trehalose biosensors. (**a**) Heat map, generated using MATLAB (Mathworks), identifying functional trehalose biosensors from a representative *in vivo* 96-well plate assay. Samples were grown for two hours post-IPTG induction and then 1 mM trehalose added. The normalized changes in fluorescence before and after trehalose addition were calculated. Wells are pseudocolored with a minimum normalized $\Delta F/F$ of 0.25 (blue) and a maximum of 0.5 (pink). Normalized $\Delta F/F \geq 0.5$ identified positive hits. Samples A1, A2 and A3 are controls. A1 – media, A2 – TMBP, A3 – cpGFP. (**b**) DNA isolated from samples in (a), were sequenced and a plot of the normalized $\Delta F/F$ values versus the insertion site generated. Data are mean ± s.d. for three biological replicates (except Tre-217 which has only two replicates).

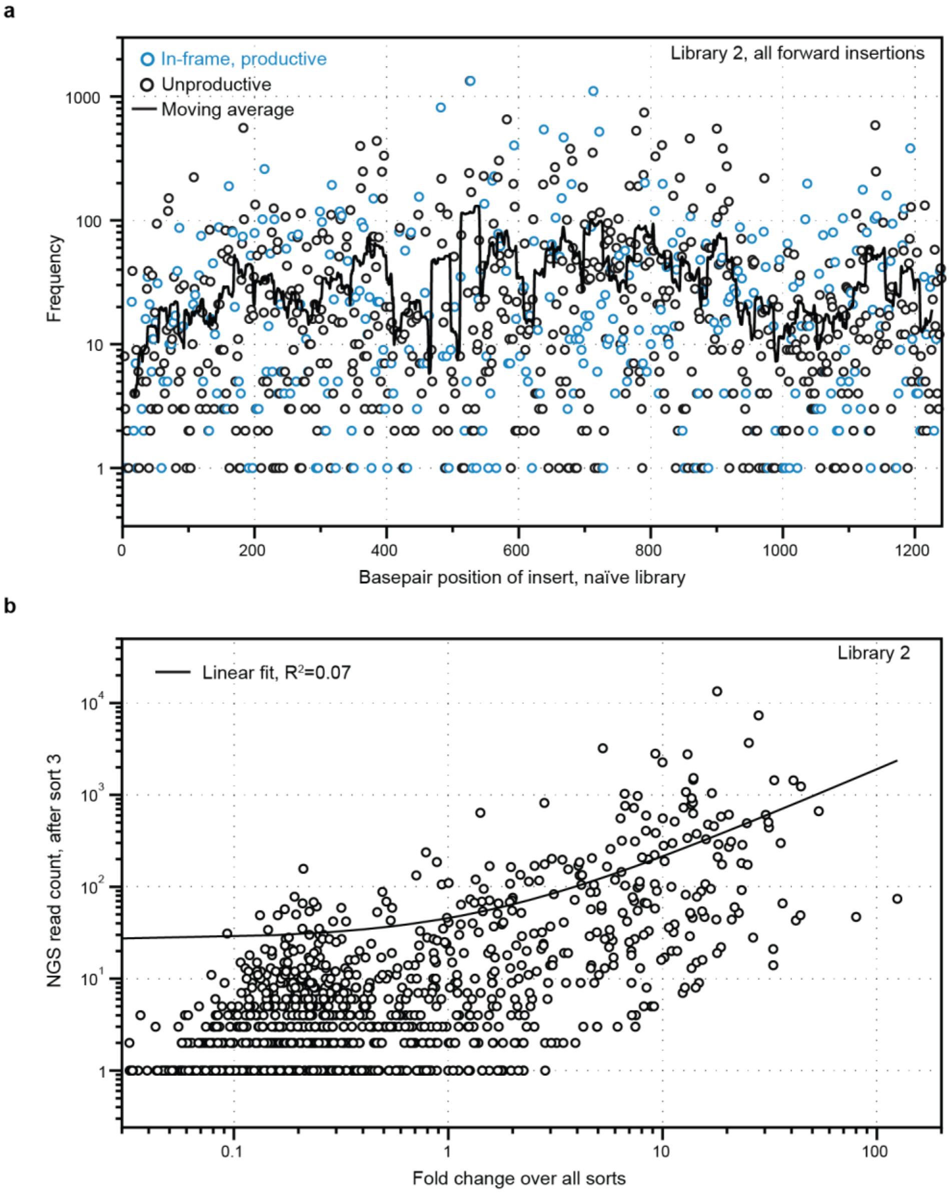

Supplementary Figure 7: TMBP-cpGFP library construct frequency. (**a**) Representative initial library distribution of NGS read counts. Includes all forward insertions, with in-frame (productive) insertions shown in blue and out-of-frame (unproductive) insertions shown in black. A moving average is shown for all positions. (**b**) NGS read count versus enrichment after three rounds of FACS. Data is from one representative biological replicate. NGS read count is from the final library, after three sorts. Fold change is calculated from the read counts of the final library compared to those from the initial library. All insertion positions and directions that were detectable (both productive and unproductive) are shown. A linear fit, calculated with from all data points, is shown.

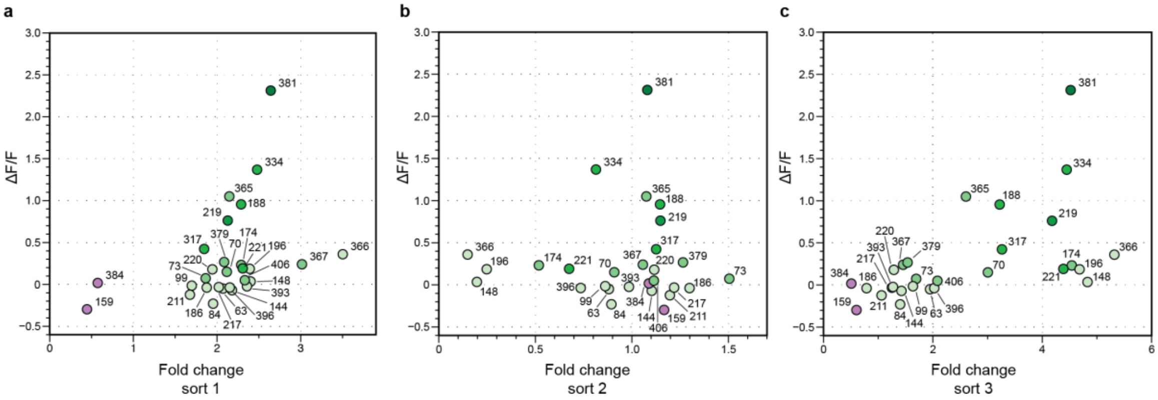

Supplementary Figure 8: Activity versus individual sort enrichments. Activity shown is the same data from Figure 4a-b. Error of activity values is left off for clarity. Enrichment, shown as fold change, is calculated with DESeq from two biological replicates comparing NGS read counts post-sort to read counts pre-sort. All constructs from Figure 4a-b are shown, regardless of calculated-enrichment P-value for the given sort, to display overall trends. (**a**) Enrichment from first sort (positive sort, with trehalose). (**b**) Enrichment from second sort (negative sort, without trehalose). (**c**) Enrichment from third (final) sort (positive sort, with trehalose). Colors of points in (a-c) are binned based on enrichment (fold change) over all sorts (see x-axis in Figure 4b).

a

TMBP insert site	ΔF/F
381	2.31
334	1.37
365	1.05
188	0.95
219	0.76
317	0.42
366	0.36
373	0.32
379	0.27
367	0.24
174	0.23

b

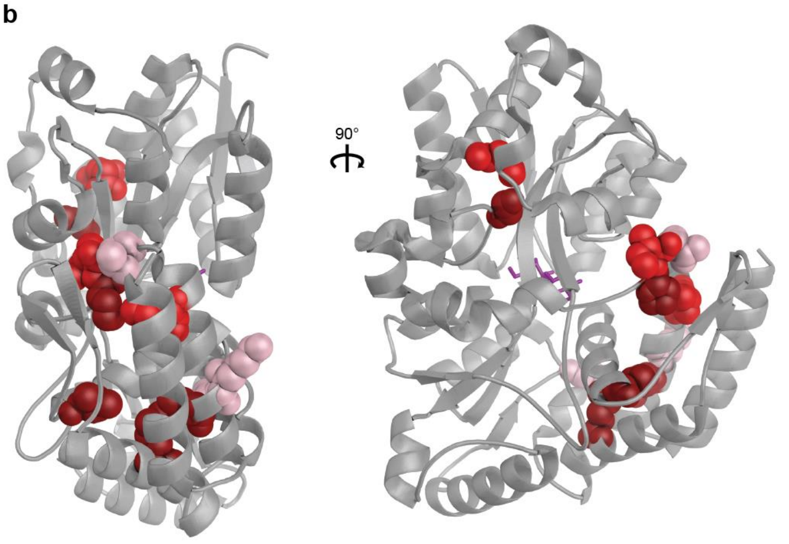

c

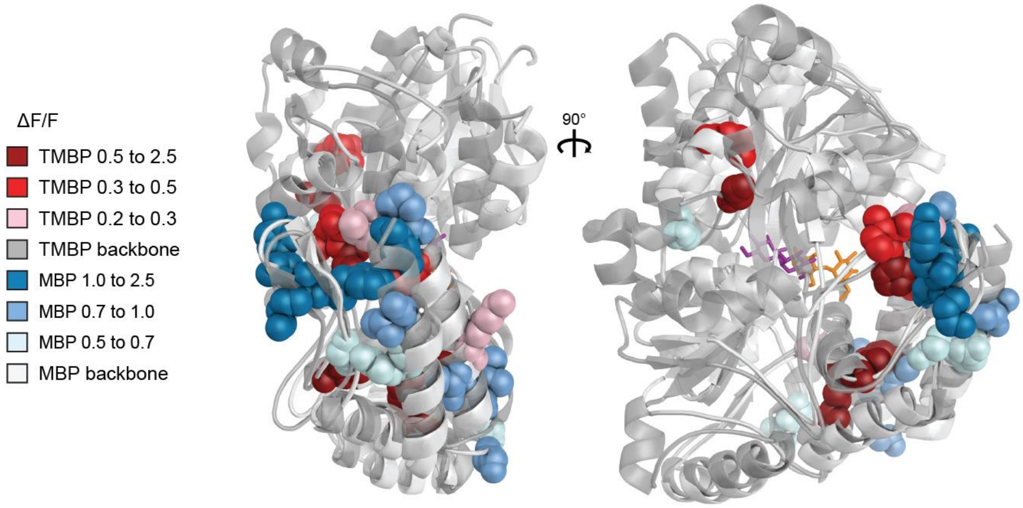

Supplementary Figure 9: Functional sites for TMBP-cpGFP biosensor constructs. (**a**) Table of top activity measurements for different insertion positions. Activity (ΔF/F) values are those from Fig 4a. (**b**) Insertion positions of top biosensors mapped on TMBP structure (1EU8). Highlighted sites are colored based on bins in (a) and are shown with sphere representations of their side chains. Bound trehalose is shown in purple. Note that, unlike MBP, TMBP only has a solved crystal structure for its trehalose-bound form. (**c**) Structural alignment of TMBP (gray) and MBP (white), both in ligand-bound forms, with highlighted functional insertion sites. Alignment carried out in PyMOL and only uses residues near the clustered hotspots to more accurately show proximity. TMBP sites use bin colors shown in (a). MBP sites use bin values shown in supplementary Figure 2c, but with blue instead of red coloring. Bound maltose is shown in orange and bound trehalose shown in purple.

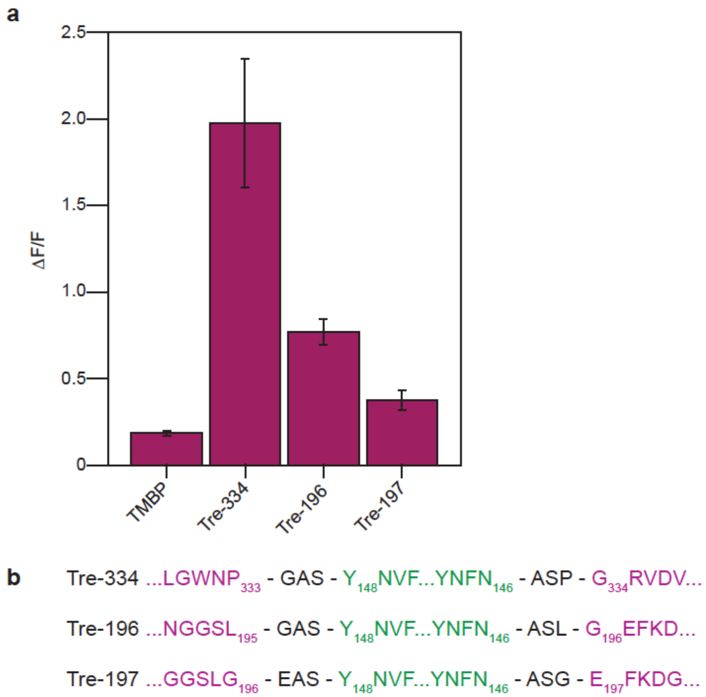

Supplementary Figure 10: Construction of the homologous Mal-170 site in TMBP does not produce a good trehalose biosensor. (**a**) The fluorescence response of Tre-196 and Tre-197 to 1 mM trehalose in an *in vivo* plate assay compared to a TMBP control and the isolated biosensor Tre-334. Data are mean ± s.d. for three replicates. (**b**) Sequence of amino acids in the linker region of Tre-334, Tre-196 and Tre-197.

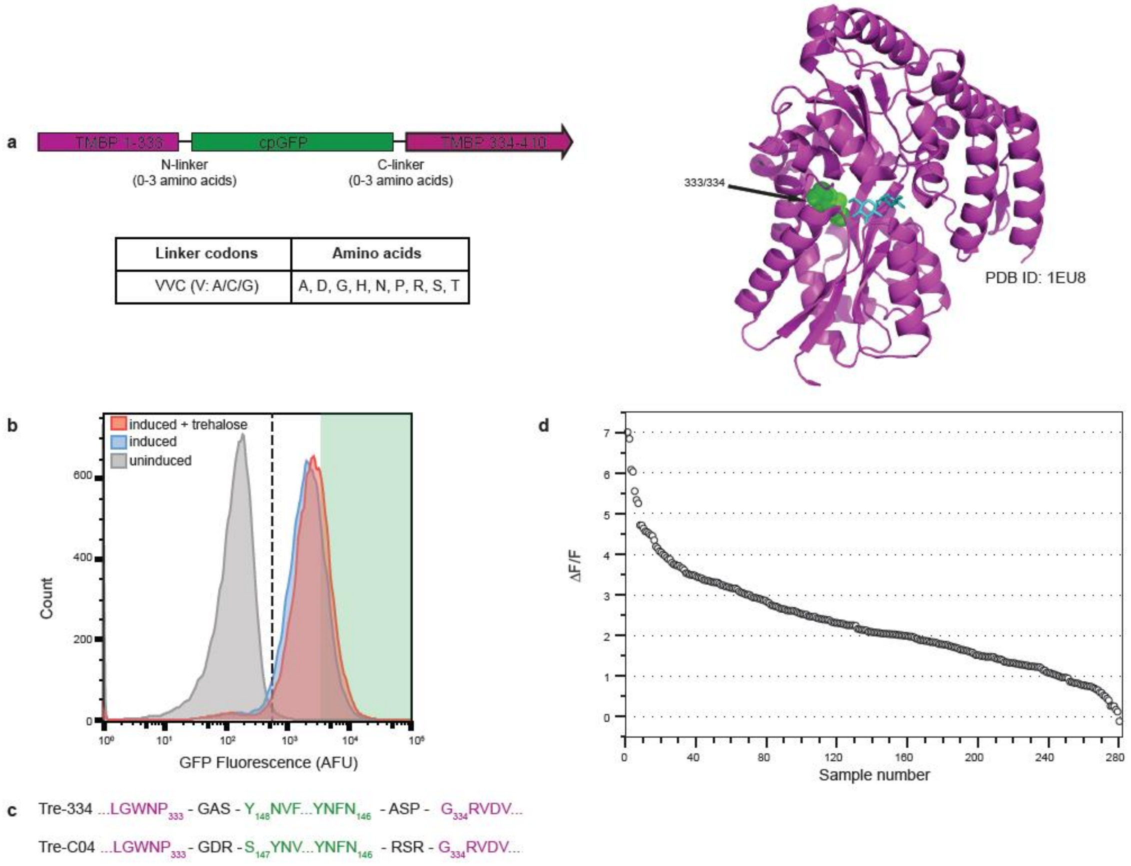

Supplementary Figure 11: Linker optimization increases the dynamic range of Tre-334. (**a**) A zero to three amino acid linker library (encoded by VVC codon) was created between amino acids 333 and 334 of TMBP as shown in the schematic and the PDB structure (PDB ID: 1EU8). The VVC codon provides equal representation of 9 amino acids as indicated. (**b**) Changes in the fluorescence histograms of the amino acid linker library upon IPTG induction and 1 mM trehalose addition. 25,000 events are shown. The dashed line indicates events that are at a non-fluorescent sample threshold and the shaded region indicates the gate for sorted events in the induced sample treated with trehalose. (**c**) The linker sequences of the parental and optimized trehalose biosensors, Tre-334 and Tre-C04 are shown. (**d**) The $\Delta F/F$ value of three 96-well plates tested in an *in vivo* assay for trehalose sensing. Samples were grown for two hours post-IPTG induction and then 1 mM trehalose added. $\Delta F/F$ values were calculated from the changes in fluorescence before and after trehalose addition.

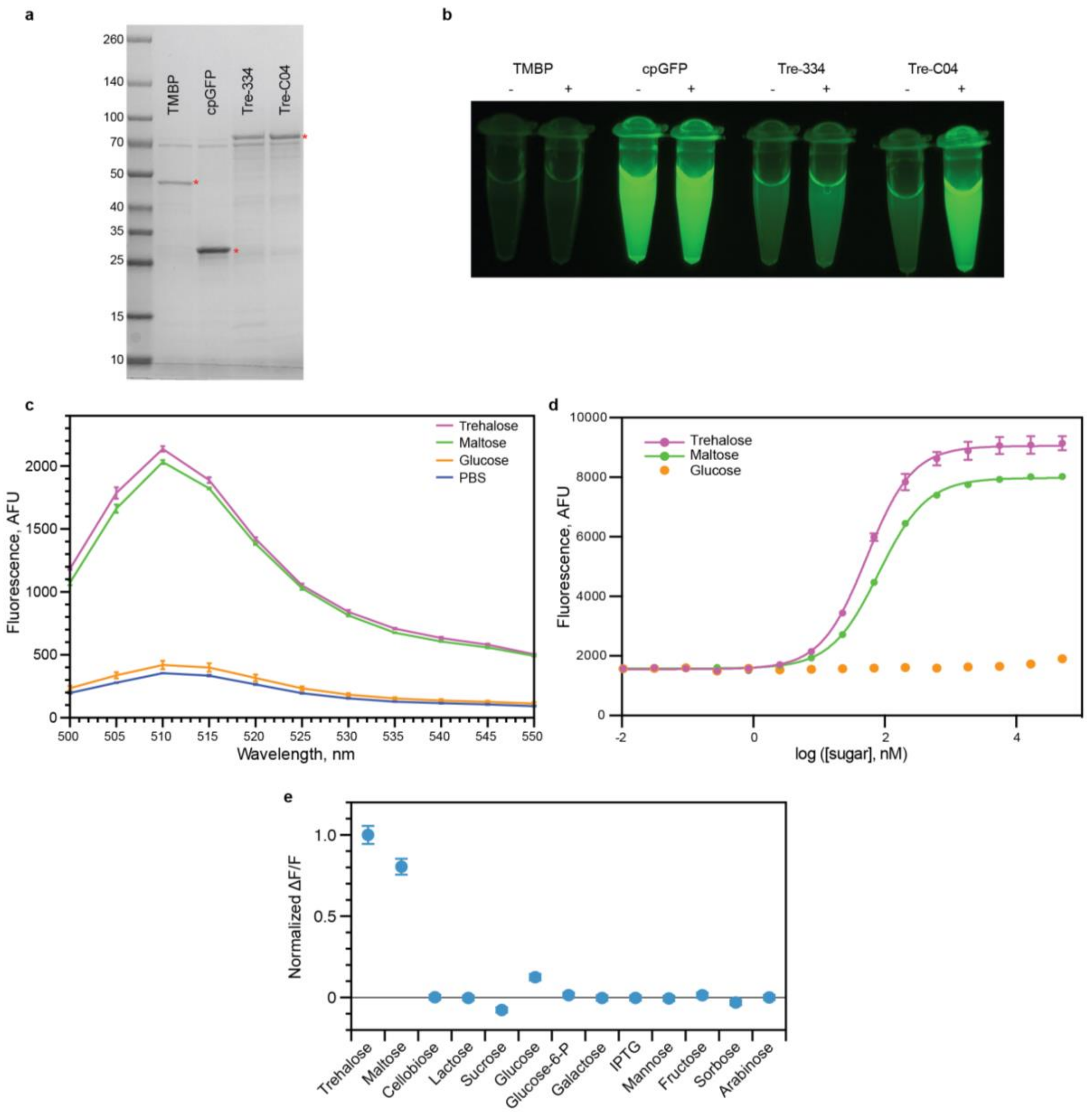

Supplementary Figure 12: Tre-C04 functions well *in vitro*. (**a**) SDS-PAGE of expressed, Ni-NTA-purified proteins, as indicated. (**b**) Pseudocolored fluorescence image of 1 μM purified protein, as indicated, in the absence or presence of 1 mM trehalose. (**c**) Fluorescence spectrum of purified, optimized trehalose biosensor, Tre-C04, with PBS or added sugar. Data are mean ± s.d. for three samples. (**d**) Fluorescence dose-response curves of purified, optimized trehalose biosensor, Tre-C04, to trehalose, maltose, and glucose. Data are mean ± s.d. for three replicates. Apparent K_d = 53 ± 1 nM (trehalose) and 81 ± 1 nM (maltose). (**e**) Biosensor response measurements (ΔF/F normalized to trehalose response) of Tre-C04 with sugar substrates (1 mM). Data are mean ± s.d. for three replicates.

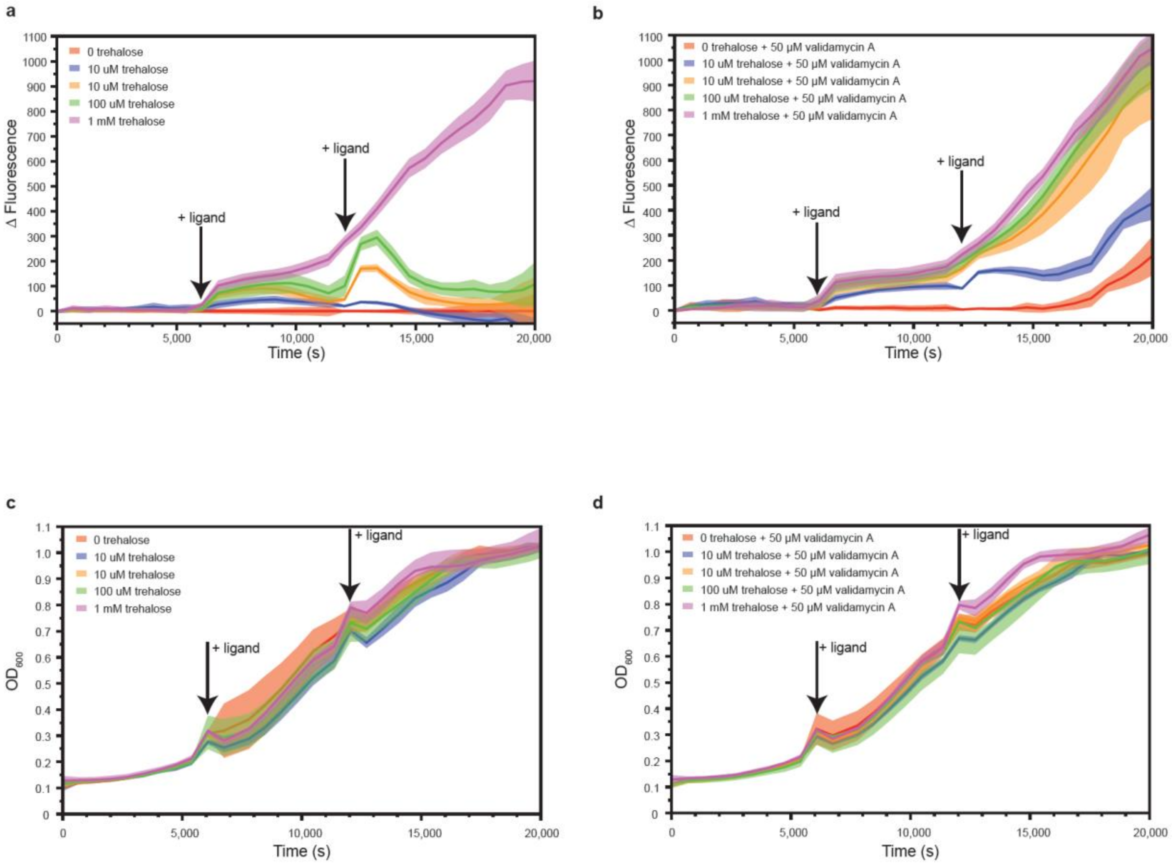

Supplementary Figure 13: Validamycin A treatment of cultures expressing Tre-C04 results in a sustained fluorescence response. (**a**) Fluorescence reponse of the optimized biosensor, Tre-C04, to 0, 1 µM, 10 µM, 100 µM and 1 mM trehalose. Fluorescence was background corrected against a culture treated with water instead of trehalose. (**b**) Fluorescence reponse of the optimized biosensor, Tre-C04, to 50 µM validamycin A and 0, 1 µM, 10 µM, 100 µM or 1 mM trehalose. Fluorescence was background corrected against a culture treated with water instead of trehalose. (**c**) OD_{600} for samples shown in (a). (**d**) OD_{600} for samples shown in (b). For (**a**-**d**), arrows indicate additions and data are mean ± s.d. for twelve replicates.

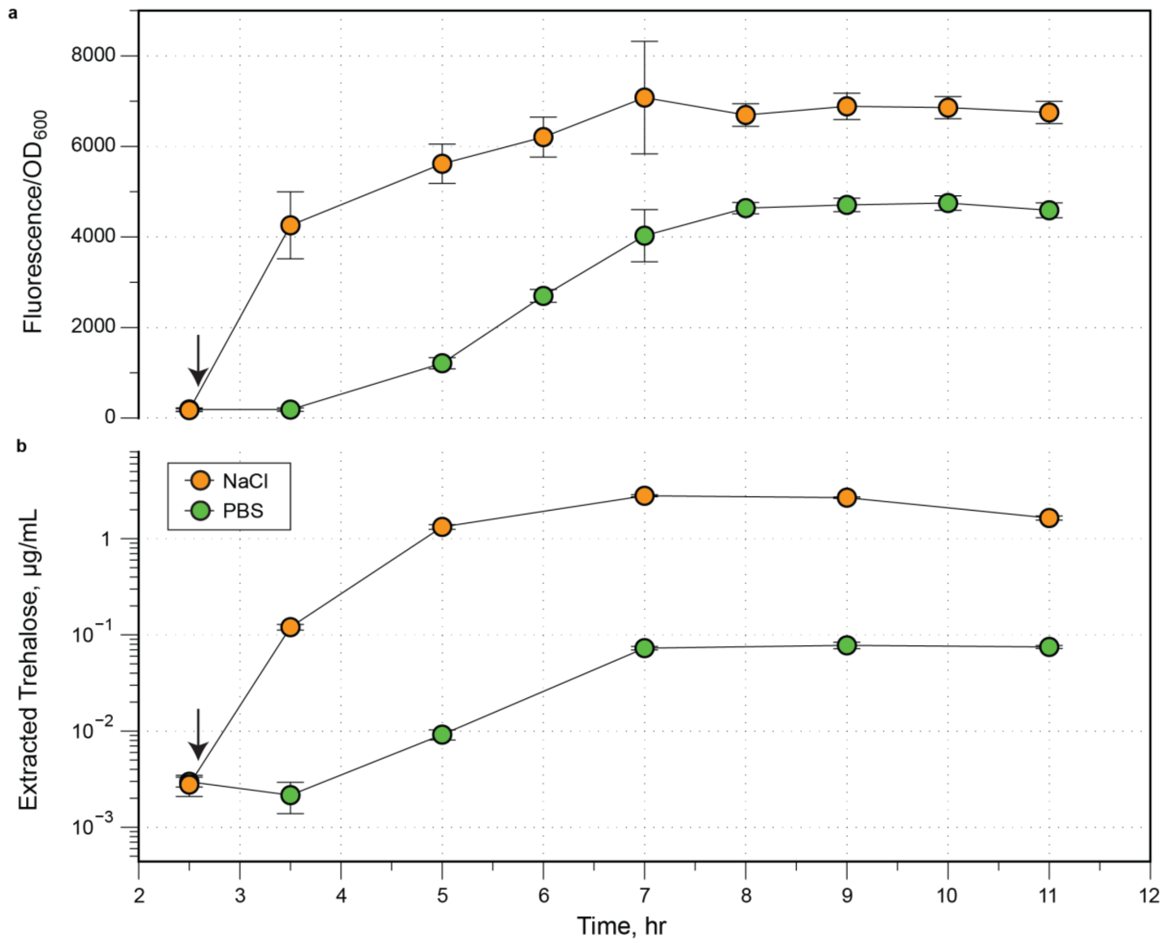

Supplementary Figure 14: Extraction and measurement of free sugars during *in vivo* NaCl exposure. (**a**) Fluorescence response of Tre-C04 to the addition of PBS or 300 mM NaCl (final concentration). Arrow indicates time of additions. Data are mean ± s.d. for three biological replicates. (**b**) Concentration of trehalose in cell extracts from cultures in (a) measured by HPAEC-PAD analysis. Data are mean ± s.d. for three biological replicates. No maltose was detected for all culture conditions and time points (limit of detection ~0.05 µg/mL).

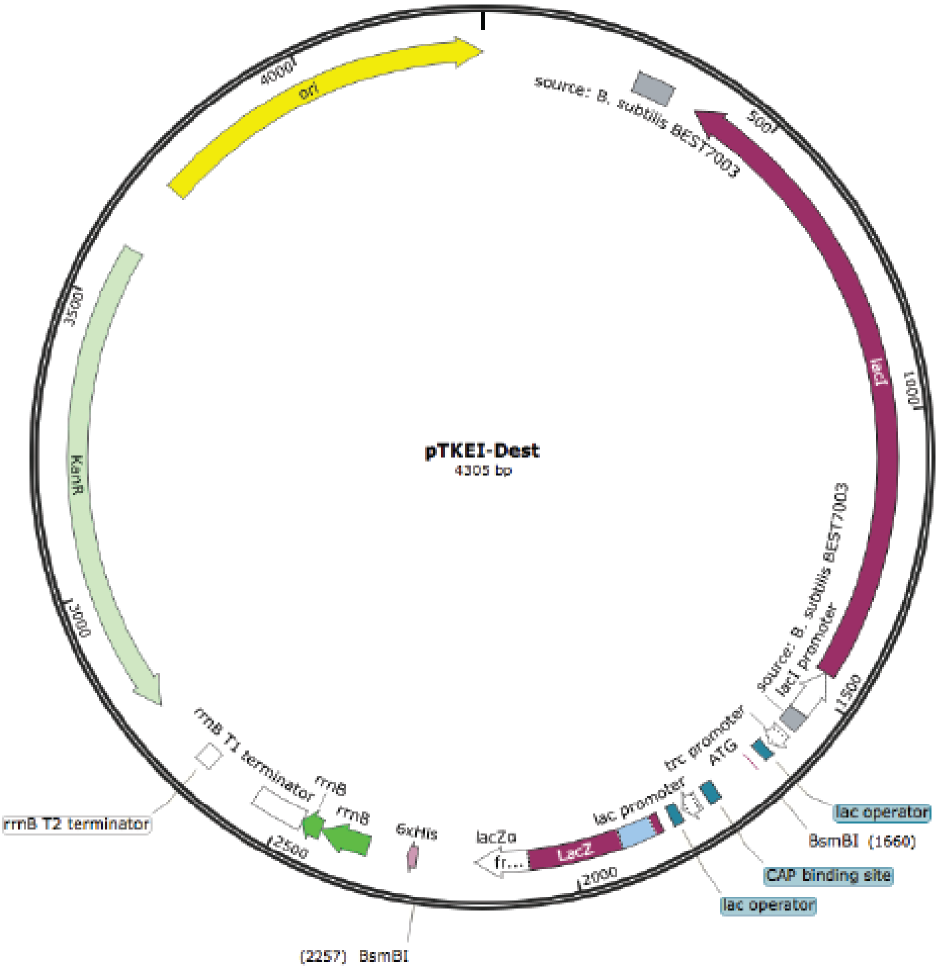

Supplementary Figure 15: pTKEI-Dest plasmid map. Generated with SnapGene software (GSL Biotech).

Supplementary Table 1: DNA sequences. For primers, lower case letters indicate bases that anneal to the template

Type	Name	Sequence
ORF	MBP	AAAATCGAAGAAGGTAAACTGGTAATCTGGATTAACGGCGATAAAGGCTATAACGGACTCGCTGAAGTCGGTAAGAAATTCGAGAAAGATACCGGAATTAAAGTCACCGTTGAGCATCCGGATAAACTGGAAGAGAAATTCCCACAGGTTGCGGCAACTGGCGATGGCCCTGACATTATCTTCTGGGCACACGACCGCTTTGGTGGCTACGCTCAATCTGGCCTGTTGGCTGAAATCACCCCGGACAAAGCGTTCCAGGACAAGCTGTATCCGTTTACCTGGGATGCCGTACGTTACAACGGCAAGCTGATTGCTTACCCGATCGCTGTTGAAGCGTTATCGCTGATTTATAACAAAGATCTGCTGCCGAACCCGCCAAAAACCTGGGAAGAGATCCCGGCGCTGGATAAAGAACTGAAAGCGAAAGGTAAGAGCGCGCTGATGTTCAACCTGCAAGAACCGTACTTCACCTGGCCGCTGATTGCTGCTGACGGGGGTTATGCGTTCAAGTATGAAAACGGCAAGTACGACATTAAAGACGTGGGCGTGGATAACGCTGGCGCGAAAGCGGGTCTGACCTTCCTGGTTGACCTGATTAAAAACAAACACATGAATGCAGACACCGATTACTCCATCGCAGAAGCTGCCTTTAATAAAGGCGAAACAGCGATGACCATCAACGGCCCGTGGGCATGGTCCAACATCGACACCAGCAAAGTGAATTATGGTGTAACGGTACTGCCGACCTTCAAGGGTCAACCATCCAAACCGTTCGTTGGCGTGCTGAGCGCAGGTATTAACGCCGCCAGTCCGAACAAAGAGCTGGCGAAAGAGTTCCTCGAAAACTATCTGCTGACTGATGAAGGTCTGGAAGCGGTTAATAAAGACAAACCGCTGGGTGCCGTAGCGCTGAAGTCTTACGAGGAAGAGTTGGCGAAAGATCCACGTATTGCCGCCACCATGGAAAACGCCCAGAAAGGTGAAATCATGCCGAACATCCCGCAGATGTCCGCTTTCTGGTATGCCGTGCGTACTGCGGTGATCAACGCCGCCAGCGGTCGTCAGACTGTCGATGAAGCCCTGAAAGACGCGCAGACTCGTATCACCAAG
ORF	TMBP	AAAATCGAGGAAGGTAAAATTGTGTTTGCAGTGGGTGGCGCCCCGAATGAAATCGAATATTGGAAAGGTGTTATTGCCGAATTCGAGAAAAAATATCCTGGGGTTACAGTGGAGCTGAAACGCCAGGCCACCGATACCGAACAGCGTCGTCTGGATCTGGTTAATGCGCTTCGCGGGAAAAGCTCCGACCCTGATGTATTCTTGATGGACGTCGCGTGGCTGGGTCAGTTCATCGCTTCTGGTTGGCTGGAACCCCTGGACGATTATGTTCAAAAAGACAATTATGACCTGAGCGTGTTCTTTCAGTCGGTGATTAACCTTGCGGACAAACAGGGCGGCAAACTGTATGCCCTGCCGGTGTATATTGATGCAGGTCTGCTGTACTATCGTAAAGATCTTCTTGAAAAATACGGCTATTCTAAGCCGCCGGAAACATGGCAGGAACTGGTCGAGATGGCCCAAAAAATTCAGTCGGGGGAGCGCGAAACCAACCCAAATTTTTGGGGTTTTGTTTGGCAGGGCAAACAATACGAGGGCCTGGTGTGCGACTTCGTGGAGTACGTGTACTCAAACGGCGGAAGCTTAGGTGAATTTAAAGACGGCAAGTGGGTCCCGACTTTGAACAAACCGGAAAATGTTGAGGCCCTGCAATTCATGGTGGATCTGATCCACAAGTATAAGATTTCGCCTCCAAACACCTACACGGAAATGACGGAAGAACCGGTACGCCTGATGTTTCAACAGGGCAACGCGGCGTTCGAGCGTAATTGGCCATATGCTTGGGGCCTGCATAATGCTGACGATTCTCCAGTGAAAGGCAAAGTCGGTGTGGCCCCGTTGCCACATTTTCCGGGCCATAAAAGCGCGGCTACCCTGGGTGGTTGGCACATTGGTATTAGCAAGTATAGCGACAATAAAGCCTTGGCGTGGGAATTTGTGAAATTCGTAGAAAGTTATAGCGTCCAGAAAGGATTTGCGATGAATTTGGGTTGGAATCCCGGGCGCGTAGACGTATACGACGATCCGGCGGTGGTGTCCAAAAGCCCCCATCTGAAAGAGCTGCGTGCGGTGTTTGAAAACGCGGTCCCACGCCCAATCGTACCGTATTACCCGCAACTGAGTGAAATCATCCAGAAATACGTTAACTCCGCCCTTGCAGGCAAAATCTCCCCGCAGGAAGCACTGGATAAAGCTCAAAAAGAAGCCGAAGAACTGGTAAAGCAGTATTCA
ORF	cpEGFP	TATAACGTCTTTATCATGGCCGACAAGCAGAAGAACGGCATCAAGGCGAACTTCAAGATCCGCCACAACATCGAGGACGGCGGCGTGCAGCTCGCCTATCACTACCAGCAGAACACCCCCATCGGCGACGGCCCCGTGCTGCTGCCCGACAACCACTACCTGAGCGTGCAGTCCAAACTGAGCAAAGACCCCAACGAGAAGCGCGATCACATGGTCCTGCTGGAGTTCGTGACCGCCGCCGGGATCACTCTCGGCATGGACGAGCTGTACAAGGGCGGTACCGGAGGGAGCATGGTGAGCAAGGGCGAGGAGCTGTTCACCGGGGTGGTGCCCATCCTGGTCGAGCTGGACGGCGACGTAAACGGCCACAAGTTCAGCGTGTCCGGCGAGGGCGAGGGCGATGCCACCTACGGCAAGCTGACCCTGAAGTTCATCTGCACCACCGGCAAGCTGCCCGTGCCCTGGCCCACCCTCGTGACCACCCTGACCTACGGCGTGCAGTGCTTCAGCCGCTACCCCGACCACATGAAGCAGCACGACTTCTTCAAGTCCGCCATGCCCGAAGGCTACATTCAGGAGCGCACCATCTTCTTCAAGGACGACGGCAACTATAAGACACGCGCTGAGGTTAAGTTCGAGGGCGACACTCTGGTTAACCGCATCGAGCTGAAGGGCATCGACTTCAAGGAGGACGGCAACATCCTGGGCCATAAGCTTGAATATAACTTCAAC
primer	entranceposon_Ins1_F	CACACCAGGTCTCAGTCCCCAGAGGATTAgatctgAAgcGgcgcacgaaaaacg
primer	entranceposon_Ins1_R	CACACCAGGTCTCAAtctcattttcgccaaaag
primer	entranceposon_Ins2_F	CACACCAGGTCTCAagaTgttgatcggcacgtaagag
primer	entranceposon_Ins2_R	CACACCAGGTCTCAACacgaaaaacatattctcaataaacc
primer	entranceposon_Ins3_F	CACACCAGGTCTCAgtGtcagccaatccctgg
primer	entranceposon_Ins3_R	CACACCAGGTCTCACGCTATACATAGCAAAGCTTGAAgcGgcgcacgaaaaacg
primer	CmR_int_R_bsai	CACACCAGGTCTCAcgtaacacgccacatcttgcg
primer	CmR_int_F_bsai	CACACACGGTCTCAtacggtgaaaacctggcctatttcc
primer	CmR_int_R_bsmbi	CACACCACGTCTCAcgtaacacgccacatcttgcg
primer	CmR_int_F_bsmbi	CACACACCGTCTCAtacggtgaaaacctggcctatttcc
primer	Mu-BsrDI-GG-F	CACACACGGTCTCAGTCCagatctgCaTTgCcgcacgaaaaacgcgaaagc

primer	Mu-BsrDI-GG-R	CACACCAGGTCTCACGCTaagcttgCaTTgCcgcacgaaaaacgcgaaagc
primer	Mu-BtsI-GG-F	CACACACGGTCTCAGTCCagatctgaCAcTgcgcacgaaaaacgcgaaag
primer	Mu-BtsI-GG-R	CACACCAGGTCTCACGCTaagcttgaCAcTgcgcacgaaaaacgcgaaag
primer	Mu-EarI-GG-F	CACACACGGTCTCAGTCCagatctgaagAAgAgcacgaaaaacgcgaaagcg
primer	Mu-EarI-GG-R	CACACCAGGTCTCACGCTaagcttgaagAAgAgcacgaaaaacgcgaaagcg
primer	Mu-BsaI-2-GG-F	CACACACCGTCTCAGTCCagatctgaagGAgACcacgaaaaacgcgaaagcgtttc
primer	Mu-BsaI-2-GG-R	CACACCACGTCTCACGCTaagcttgaagGAgACcacgaaaaacgcgaaagcgtttc
primer	Mu-BsmBI-1-GG-F	CACACCAGGTCTCAGTCCagatctgaagGAgACGacgaaaaacgcgaaagcgtttcac
primer	Mu-BsmBI-1-GG-R	CACACACGGTCTCACGCTaagcttgaagGAgACGacgaaaaacgcgaaagcgtttcac
primer	Mu-BsmBI-2-GG-F	CACACCAGGTCTCAGTCCagatctgaagcggGAGacgaaaaacgcgaaagcgtttcac
primer	Mu-BsmBI-2-GG-R	CACACACGGTCTCACGCTaagcttgaagcggGAGacgaaaaacgcgaaagcgtttcac
primer	Mu-BsaI-1-GG-F	CACACCACGTCTCAGTCCagatctgaagcggAgACcgaaaaacgcgaaagcgtttcac
primer	Mu-BsaI-1-GG-R	CACACACCGTCTCACGCTaagcttgaagcggAgACcgaaaaacgcgaaagcgtttcac
primer	Mu-BsmBI-3-GG-F	CACACACGGTCTCAGTCCagatctgaagcgAGAcGcgaaaaacgcgaaagcgtttcac
primer	Mu-BsmBI-3-GG-R	CACACCAGGTCTCACGCTaagcttgaagcgAGAcGcgaaaaacgcgaaagcgtttcac
primer	Mu-BpiI-GG-F	CACACACGGTCTCAGTCCagatctgaagcgTcTTCcgaaaaacgcgaaagcgtttcac
primer	Mu-BpiI-GG-R	CACACCAGGTCTCACGCTaagcttgaagcgTcTTCcgaaaaacgcgaaagcgtttcac
primer	BsaI-M1-Cmr-1-GG-F	CACACCACGTCTCAGTCCagatctgCaTcggAgACcgaaaaacgcgaaagcgtttcac
primer	BsaI-M1-Cmr-1-GG-R	CACACACCGTCTCACGCTaagcttgaCgcggAgACcgaaaaacgcgaaagcgtttcac
primer	cpEGFP-M1-GG-F	CACACCAGGTCTCACATCTtataacgtctttatcatggccgacaagc
primer	cpEGFP-M1-GG-R	CACACCAGGTCTCTACGCgttgaagttatattcaagcttatggcccag
primer	cpEGFP-MBP170-F0	CACACCAGGTCTCAGTTCtataacgtctttatcatggccgacaagc
primer	cpEGFP-MBP170-F1	CACACCAGGTCTCAGTTCVSTtataacgtctttatcatggccgacaagc
primer	cpEGFP-MBP170-F2	CACACCAGGTCTCAGTTCVSTVSTtataacgtctttatcatggccgacaagc
primer	cpEGFP-MBP170-F3	CACACCAGGTCTCAGTTCVSTVSTVSTtataacgtctttatcatggccgacaagc
primer	cpEGFP-MBP170-F4	CACACCAGGTCTCAGTTCVSTVSTVSTVSTtataacgtctttatcatggccgacaagc
primer	cpEGFP-MBP170-R0	CACACCAGGTCTCTACTTgttgaagttatattcaagcttatggcccag
primer	cpEGFP-MBP170-R1	CACACCAGGTCTCTACTTASBgttgaagttatattcaagcttatggcccag
primer	cpEGFP-MBP170-R2	CACACCAGGTCTCTACTTASBASBgttgaagttatattcaagcttatggcccag
primer	cpEGFP-MBP170-R3	CACACCAGGTCTCTACTTASBASBASBgttgaagttatattcaagcttatggcccag
primer	cpEGFP-MBP170-R4	CACACCAGGTCTCTACTTASBASBASBASBgttgaagttatattcaagcttatggcccag
primer	MBP-170-F	CACACCAGGTCTCTaagtatgaaaacggcaagtacgacattaaagac
primer	MBP-170-R	CACACCAGGTCTCAgaacgcataacccccgtcag
primer	Tre-333/334-F0	CACACCAGGTCTCATCCGtcttataacgtctttatcatggc
primer	Tre-333/334-F1	CACACCAGGTCTCATCCGVVCtcttataacgtctttatcatggc
primer	Tre-333/334-F2	CACACCAGGTCTCATCCGVVCVVCtcttataacgtctttatcatggc
primer	Tre-333/334-F3	CACACCAGGTCTCATCCGVVCVVCVVCtcttataacgtctttatcatggc
primer	Tre-333/334-R0	CACACCAGGTCTCAGACCGttgaagttatattcaagcttatggc
primer	Tre-333/334-R1	CACACCAGGTCTCAGACCGBBGttgaagttatattcaagcttatggc
primer	Tre-333/334-R2	CACACCAGGTCTCAGACCGBBGBBGttgaagttatattcaagcttatggc
primer	Tre-333/334-R3	CACACCAGGTCTCAGACCGBBGBBGBBGttgaagttatattcaagcttatggc
primer	Tre-G334-F	CACACCAGGTCTCAGGTcgcgtagacgtatacgac
primer	Tre-P333-R	CACACCAGGTCTCACggattccaacccaaattcatcgc
